# Shear-enhanced liquid-crystal spinning of conjugated polymer fibers

**DOI:** 10.1093/nsr/nwaf331

**Published:** 2025-08-13

**Authors:** Hao Jiang, Chi-Yuan Yang, Deyu Tu, Yueheng Zhong, Zhu Chen, Wei Huang, Liang-Wen Feng, Hengda Sun, Christian Müller, Antonio Facchetti, Hongzhi Wang, Simone Fabiano, Gang Wang

**Affiliations:** State Key Laboratory of Advanced Fiber Materials, College of Materials Science and Engineering, Donghua University, Shanghai 201620, China; Laboratory of Organic Electronics, Department of Science and Technology, Linköping University, Norrköping SE-60174, Sweden; Laboratory of Organic Electronics, Department of Science and Technology, Linköping University, Norrköping SE-60174, Sweden; State Key Laboratory of Advanced Fiber Materials, College of Materials Science and Engineering, Donghua University, Shanghai 201620, China; Key Laboratory of Green Chemistry & Technology, Ministry of Education, College of Chemistry, Sichuan University, Chengdu 610065, China; School of Automation Engineering, University of Electronic Science and Technology of China, Chengdu 611731, China; Key Laboratory of Green Chemistry & Technology, Ministry of Education, College of Chemistry, Sichuan University, Chengdu 610065, China; State Key Laboratory of Advanced Fiber Materials, College of Materials Science and Engineering, Donghua University, Shanghai 201620, China; Department of Chemistry and Chemical Engineering, Chalmers University of Technology, Göteborg 41296, Sweden; School of Materials Science and Engineering, Georgia Institute of Technology, Atlanta, GA 30332, USA; State Key Laboratory of Advanced Fiber Materials, College of Materials Science and Engineering, Donghua University, Shanghai 201620, China; State Key Laboratory of Advanced Fiber Materials, College of Materials Science and Engineering, Donghua University, Shanghai 201620, China; Laboratory of Organic Electronics, Department of Science and Technology, Linköping University, Norrköping SE-60174, Sweden; State Key Laboratory of Advanced Fiber Materials, College of Materials Science and Engineering, Donghua University, Shanghai 201620, China; Yuyue Home Textile Co., Ltd, Binzhou 256623, China

**Keywords:** liquid crystal, semiconductor fiber, fabric electronics, conjugated polymer

## Abstract

Conjugated polymer fibers hold great promise for manufacturing unconventional electronic devices, particularly for advancing the applicability of wearable technology and smart textiles. For instance, these fibers have recently been used for energy conversion, electrochemical sensing and platforms for human–machine interactions. However, the limited methods available for spinning fibers from conjugated polymers with rigid backbones have impeded progress in wearable applications. Here, we report the continuous production of anisotropic semiconductor fibers by modulating π–π stacking interactions of liquid-crystalline conjugated polymers under shear stress. This method allows rigid conjugated polymers to be processed, synergistically enhancing both the mechanical and semiconductor properties of fibers through liquid-crystal spinning. As a result, these fibers exhibit excellent electrochemical performance, high mechanical strength (∼600 MPa) and outstanding scalability, as well as stability under extreme temperatures, UV radiation and chemical reagent exposure. Moreover, a fully textile-based visual logic sensing system was developed using semiconductor-fiber organic electrochemical transistors, offering a novel technological approach for integrating smart textiles into precision medicine and health monitoring. These findings underscore the importance of the liquid crystalline state and solution control in optimizing the performance of conjugated polymer fibers, paving the way for developing a new generation of fiber semiconductor devices.

## Teaser

Shear-induced liquid crystal alignment enables continuous fabrication of semiconducting polymer fibers with enhanced electrical and mechanical performance, establishing a platform for next-generation bio-interactive electronic textiles.

## INTRODUCTION

π-Conjugated polymers exhibit semiconducting behavior, making them suitable for a wide range of applications such as flexible electronics, organic photovoltaics and biosensors [1–4]. Recent developments in the design and synthesis of conjugated polymers have imparted diverse electronic functionalities to fibers and textiles, enhancing their appeal for human–machine interfaces, personalized healthcare and energy conversion [5–8]. Fiber-based electronic devices that can generate, transmit and modulate electronic functionalities are among the most promising forms of wearable electronics available today [9–12].

The processes used for the preparation of fabrics, such as weaving and knitting, often require fibers and yarns to possess sufficient strength. Fibers used for mechanized garment production should possess adequate mechanical properties, such as a yield strain (*ε*_y_) of at least several percent [13], a high yield strength (*σ*_y_) and ideally, a high tensile strength (*σ*_s_) comparable to common materials such as cotton (*σ*_s_ > 400 MPa) and polyester fiber (*σ*_s_ > 450 MPa). However, it remains challenging to obtain that degree of yield and tensile strength for fibers made from conjugated polymers while preserving excellent semiconductor behavior. The inherent backbone rigidity and high melting temperatures near thermal decomposition complicate the preparation of conjugated polymer fibers through methods like electrospinning and direct melt spinning [14–17].

In the case of wet spinning, poly(3,4-ethylenedioxythiophene):poly(styrenesulfonate) (PEDOT:PSS) and poly(benzodifurandione) (PBFDO) filaments with enhanced mechanical and electrochemical properties have been achieved through post-spinning drawing and coagulation bath treatments [18–21]. For example, PEDOT:PSS filaments with a *σ*_s_ of up to 410 MPa and *ε*_y_ of 2.5% have been demonstrated [20], while PBFDO filaments display a lower *σ*_s_ of up to 250 MPa and a low *ε*_y_ of only 1% [21]. However, during the spinning process, controlling the molecular state in the solution is equally crucial for enhancing the mechanical and functional properties of the fibers—a research area that is still underexplored in the context of conjugated polymer fibers [22].

Wet spinning of liquid-crystalline (LC) polymers is a widely used technique for producing high-performance fibers, including polyaramid materials such as Kevlar^®^. This technique leverages the ordered phases of LC polymers to enhance fiber properties [23,24]. Similarly, due to the planar and rigid nature of some conjugated polymers, these materials exhibit liquid-crystal phenomena enhanced by π−π interactions [25]. The ordered aggregate structure formed in solution is preserved during the liquid-to-solid transition and thus persists in the solid-state fiber [26]. Applying fluid shear stress to the liquid-crystal aggregates can further enhance the π−π stacking of macromolecular backbones in fibers, reducing internal flaws and facilitating high orientation and crystallinity [27]. This potentially improves the charge transport and mechanical properties of conjugated polymer fibers. However, the transition of lyotropic liquid-crystal polymers from isotropic dispersions to liquid-crystal dispersions is driven by changes in concentration [28]. Despite the potential benefits of LC spinning for conjugated polymers, their commonly lower solubility makes it difficult to create LC solutions, posing a challenge in developing suitable LC spinning processes for these materials [29].

Herein, we employed fluid shear stress to achieve continuous liquid-crystal spinning of several typical conjugated polymers by promoting π−π stacking interactions, resulting in semiconductor fibers with high orientation and crystallinity. Using a combination of X-ray diffraction and electrochemical analysis, we elucidate the influence of shear stress on π−π interactions, crystallinity and charge transport characteristics of these fibers. Our findings reveal that the shear-enhanced orientation of liquid-crystal molecules leads to significant uniaxial orientation in the conjugated semiconductor fibers. This results in anisotropic electrochemical properties, with axial carrier mobility and transconductance enhanced by ∼400% compared to the radial direction. The conjugated semiconductor fibers exhibit exceptional electrochemical properties, and also possess promisingly high yield strain and strength (*ε*_y_ = 3.4% and *σ*_y_ = 383 MPa), tensile strength (*σ*_s_ ∼ 600 MPa) for textile manufacturing, and stability under extreme temperatures (−196°C to 500°C), UV radiation, and acidic or basic conditions. These attributes indicate their potential to create practical and highly adaptable logic fabrics. Furthermore, we demonstrated a large-scale, fabric-based organic electrochemical transistor (OECT) array that seamlessly integrates into textile workflows, offering real-time, non-invasive sensing (e.g. for chronic health monitoring) and paving the way toward robust, wearable smart textiles.

## RESULTS AND DISCUSSION

### Continuous liquid-crystal spinning of semiconductor fibers

**Figure 1.**
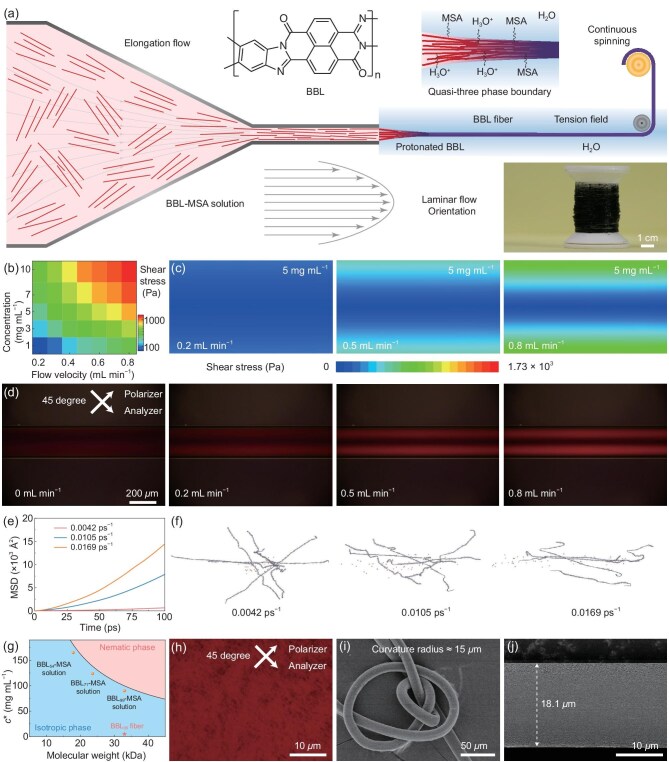
Continuous liquid-crystal spinning of semiconductor fibers. (a) Schematic of the fabrication process and photograph of macroscopic BBL_99_ semiconductor fibers. (b) Thermogram showing maximum shear rates at different flow velocities and concentrations. (c) Finite element fluid simulation of shear stress distribution in a microfluidic channel (needle diameter: 200 μm) at flow rates of 0.2, 0.5 and 0.8 mL min^−1^. (d) POM images of BBL_99_–MSA solutions in a 200 μm-diameter glass capillary under pressure-driven nematic flow, recorded at 45° to the polarizer and analyzer. Flow rates: 0, 0.2, 0.5 and 0.8 mL min^−1^. (e) The mean squared displacement (MSD) of N atoms in the BBL chain under thermal motion. (f) Molecular trajectory of the BBL–MSA solutions under different shear conditions. (g) Phase diagram showing the relationship between critical concentration and molecular weight in BBL–MSA solutions at 25°C. The solid black line indicates the theoretical phase boundary fitted using the Flory rigid-rod model. Blue and pink regions correspond to the isotropic and nematic liquid-crystal phases, respectively. (h) The POM image of the 90 mg mL^−1^ BBL_99_–MSA solution taken at 45° to the analyzer. (i) Scanning electron microscopy (SEM) image of bent BBL_99_ fiber. (j) SEM image of BBL_99_ fiber-0.8.

Poly(benzimidazobenzophenanthroline) (BBL) is an example of a conjugated polymer with a rigid backbone structure. In this study, the spinning solution is pressure-driven through a microfluidic channel, where shear forces enhance the molecular orientation and thereby promote the formation of a liquid-crystal phase. The formation of this phase aligns with previous studies describing the thermotropic [30–33] or lyotropic phases [34,35] upon shearing of polymer solutions. Owing to its rigid molecular chains, which facilitate the formation of a lyotropic liquid-crystal phase [36], and its rapid proton exchange process, BBL can be continuously spun into fibers, exhibiting a high degree of uniaxial alignment (Fig. 1a, Movie S1).

We first studied the rheological behavior of BBL during the spinning process, focusing particularly on the isotropic–anisotropic phase equilibrium and shear-induced liquid-crystal formation. To this end, finite element simulations were carried out to investigate the fluid behavior of BBL_99_ (*M*_v_ = 33.0 kDa; 99 repeating units) in methanesulfonic acid [BBL_99_–methanesulfonic acid (MSA)] at various concentrations and flow velocities in the spinneret region. The results indicate that increasing the flow rate and concentration of BBL_99_–MSA solutions increases the shear forces within the flow field (Fig. 1b, Figs S1–S4, Table S1). Calculating the fluid parameters revealed a Reynolds number (Re) ranging from 0.22 to 8.80 (Table S2), confirming that the flow remains laminar (Re < 2000). Using a 5 mg mL^−1^ BBL_99_–MSA solution (dynamic viscosity = 4.737 × 10^−2^ Pa s) as an example, increasing the flow rate substantially enhances the shear forces within the fluidic field. The shear force distribution obtained through finite element simulations closely corresponds to the intensity and distribution of transmitted light observed via *in situ* polarized optical microscopy (POM) at varying flow rates (Fig. 1c and d, Fig. S5). When viewed at a 45° angle to the analyzer direction, the fluid exhibits birefringence, and the transmitted light intensity increases with the flow rate.

Subsequently, the molecular dynamics of polymer chain interactions under shear stress were investigated to elucidate how shear forces induce the formation of low-concentration liquid-crystal phases (Fig. S6). To facilitate the simulation, we selected three shear rates of 0.0042, 0.0105 and 0.0169 ps^−1^ to investigate the effect of shear force. All simulations started from the same initial state. As the shear rate increases, the slope of the mean square displacement curve of N atoms increases correspondingly (Fig. 1e), indicating that higher shear rates promote the motion of BBL molecular chains. Additionally, a larger gyration radius of N atoms and the higher radial distribution function peaks reveal the extension and aggregation of molecular chains (Fig. S7). Molecular trajectories directly show that BBL chains align in a more orderly manner and along the shear direction (Fig. 1f). Flory's molecular theory [37,38] states that the critical concentration for liquid-crystal phase formation is determined solely by the axial ratio (*x*). For rigid rod-like BBL molecules, the critical concentration depends on molecular weight (Fig. 1g). The calculated critical concentrations of BBL_99_, BBL_71_ (*M*_v_ = 23.6 kDa; 71 repeating units) and BBL_54_ (*M*_v_ = 17.9 kDa; 54 repeating units) were 99.7, 138.5 and 181.0 mg mL^−1^, respectively. According to Onsager's second-virial theory for rigid rods [39], the isotropic–nematic transition occurs when the gain in orientational entropy compensates for the excluded-volume penalty, resulting in a critical volume fraction that decreases inversely with *x*. Under shear flow, BBL chains are stretched and exhibit enhanced π–π stacking, which increases their axial ratio. Since the critical concentration is inversely related to *x*, even a modest increase can significantly lower the threshold required for phase transition under shear. Consequently, repeated shear deformation [40] induced the emergence of a distinct liquid-crystal texture, even below the critical concentration threshold (Fig. 1g and h, Fig. S10). Furthermore, to investigate the generality of the shear-enhanced liquid-crystal spinning method, we also examined the behavior of other conjugated polymer solutions under shear. The polybisbenzimidazobenzophenanthroline-dione (BBB), the glycolated polythiophene p(g2T-T), PBFDO, low molecular weight BBL_39_ (*M*_v_ = 13 kDa; 39 repeating units) and PEDOT:PSS were selected as representative conjugated polymers with flexible chains [BBB and p(g2T-T)], rigid chains (PBFDO and BBL_39_) and mixed conjugated:non-conjugated polymer systems (PEDOT:PSS) (see Figs S11–S15 for chemical structures). Polybisbenzimidazobenzophenanthroline-dione–MSA, BBL_39_–MSA, PBFDO–DMSO and PEDOT:PSS–H_2_O were adjusted to match the dynamic viscosity of the BBL_99_–MSA solution (5 mg mL^−1^) by modifying their concentrations. We also increased the dynamic viscosity of p(g2T-T)–MSA as much as possible. This adjustment allowed us to characterize these conjugated polymer solutions under the same shear conditions (Table S3). In contrast to the rigid, rod-like molecular chain structure of BBL, BBB is a flexible-chain polymer that does not form LC mesophases in solution [36]. As a result, no birefringence was observed in the BBB–MSA solution (Fig. S11). However, with increasing shear force, the intensity of transmitted light and the anisotropy of birefringence increased for the BBL_39_–MSA and PBFDO–DMSO solutions (Figs S12 and S13). In contrast, only slight birefringence was observed in the case of PEDOT:PSS–H_2_O (Fig. S14), likely due to its blend system and the short rigid main chain of PEDOT. Even at a concentration of 25 mg mL^−1^, p(g2T-T)–MSA did not reach the comparable viscosity, and no birefringence was observed (Fig. S15).

These results indicate that the pronounced birefringence observed under shear arises from the formation of a liquid-crystal phase by rigid rod-like conjugated polymers. Liquid-crystal conjugated polymer BBL_99_ forms fibers with a well-defined annular structure, capable of bending to a radius of curvature of approximately 15 μm (Fig. 1i). The resulting microfibers have diameters ranging from 16.10 ± 0.09 to 18.08 ± 0.11 μm, depending on the flow rate (Fig. 1j, Fig. S16).

### Microstructures of semiconducting fibers

**Figure 2.**
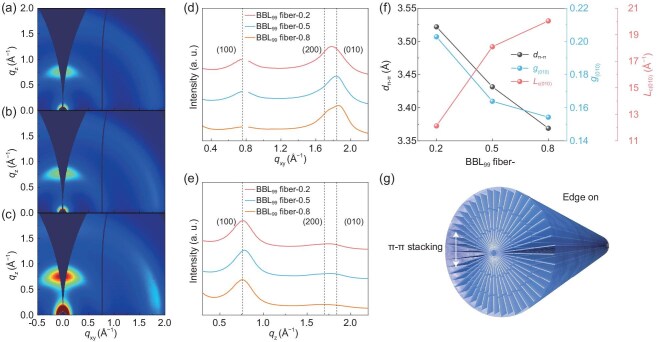
Microstructures of BBL fibers. (a–c) 2D GIWAXS patterns of BBL_99_ fiber-0.2 (a), BBL_99_ fiber-0.5 (b) and BBL_99_ fiber-0.8 (c). (d and e) In-plane (d) and out-of-plane (e) GIWAXS line profiles of BBL_99_ fibers. (f) *d*_π−π_, *L*_c(010)_ and *g*_(010)_ of BBL_99_ fibers. (g) Schematic of molecular packing in BBL_99_ fibers.

Grazing-incidence wide-angle X-ray scattering (GIWAXS) was used to characterize the microstructure of BBL_99_ fibers (Fig. 2a–c, Fig. S17). All BBL_99_ fibers exhibit a strong lamellar (100) diffraction peak at around *q*_z_ = 0.76 Å^−1^ (d-spacing = 8.27 Å) and a strong π−π stacking peak (010) at *q*_xy_ = 1.87 Å^−1^ (d-spacing = 3.37 Å) (Fig. 2d and e). As the shear effect increases during spinning, the π−π stacking distance (*d*_π−π_) decreased from 3.52 Å for BBL_99_ fiber-0.2 to 3.37 Å for BBL_99_ fiber-0.8. The full width at half maximum (FWHM) of the π–π stacking peaks for BBL_99_ fiber-0.2, -0.5 and -0.8 was 0.461, 0.309 and 0.279 Å^−1^, respectively (Fig. S18). The slight reduction in the π−π stacking distance indicates that the BBL polymer chains become more highly aligned under larger shear forces, resulting in stronger π−π interactions, longer coherence length [*L*_c(010__)_] and lower paracrystalline disorder [*g*_(010__)_] (Fig. 2f, Fig. S19) [41]. This indicates that BBL molecules adopt a highly ordered edge-on orientation aligned along the fiber axis. The molecular backbone planes are aligned parallel to the fiber axis, while π–π stacking occurs in the radial direction, perpendicular to the axial direction of the fibers (Fig. 2g). The results obtained for BBL_39_ fibers also exhibit the same trend, although the changes are relatively minor owing to the shorter backbone (Figs S20–S23).

The microstructures of BBL_99_ fiber-0.8 and films were compared using transmission wide-angle X-ray scattering (WAXS) (Fig. S24). Both BBL_99_ fibers and films show a strong π−π stacking (010) peak at around *q*_z_ = 1.87 Å^−1^ (d-spacing = 3.37 Å), while only BBL fibers show a strong lamellar (100) diffraction peak at around *q*_z_ = 0.76 Å^−1^ (d-spacing = 8.27 Å), which is consistent with the GIWAXS results. The WAXS diffractograms reveal that BBL fibers are oriented along the spinning direction due to extrusion through the nozzle, as indicated by the diffraction ring evolving into arcs. The WAXS curve at the (100) peak indicates that the Hermans’ orientation factor [42,43] of BBL_99_ fiber-0.8 is approximately 0.72. Compared to the isotropy of thin films, fibers prepared with shear-enhanced liquid-crystal phases exhibit pronounced orientation.

### Alignment, stability and mechanical properties of semiconducting fibers

**Figure 3.**
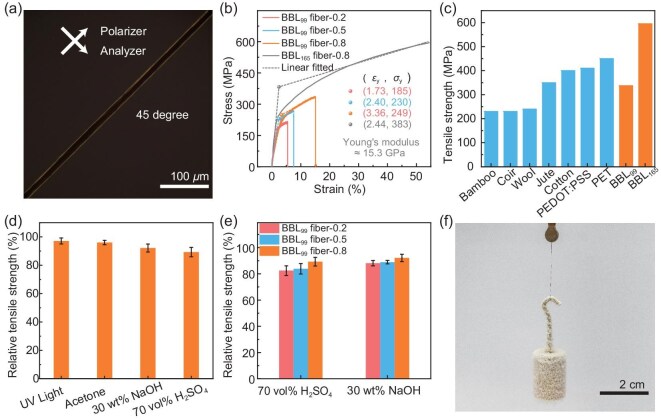
Alignment and mechanical performance of BBL fibers. (a) POM image of a BBL_99_ fiber-0.8 taken with the fiber axis at 45° relative to the polarizer and analyzer. (b) Stress–strain curves of BBL_99_ fibers and BBL_165_ fiber-0.8 measured at a strain rate of 100 mm min^−1^. (c) Tensile strength comparison between BBL_99_ fiber-0.8 (337 MPa), BBL_165_ fiber-0.8 (595 MPa) and commercial textile fibers [19, 40–42]. (d) Relative tensile strength of BBL_99_ fiber-0.8 after UV exposure and chemical treatment. (e) Relative tensile strength of BBL_99_ fibers after chemical reagent treatment. (f) Photograph of BBL_165_ fiber carrying a 10 g weight after immersion in liquid nitrogen for 3 min. Error bars represent standard deviations from three independent measurements.

The alignment of the BBL_99_ fiber-0.8 was confirmed by POM (Fig. 3a, Fig. S25). To further explore the internal structure of the fibers, we used small-angle X-ray scattering (SAXS). The 1D integration of the 2D SAXS pattern yields a scattering intensity profile as a function of *q* (Fig. S26). A distinct diffraction arc was observed at *q*_ed_ = 0.144 Å^−1^ in the equatorial direction, and it was absent in the meridian direction. This indicates the presence of a biphasic LC/isotropic structure in the BBL fiber with a periodicity of 43.8 Å [44]. Moreover, the liquid-crystal phase is aligned along the fiber axis due to the shear force.

BBL chains have planar rigid backbones that stack face-to-face, enhancing π−π interactions between polymer chains [45,46]. This increased interaction correlates with a rise in tensile strength from 218 MPa (BBL_99_ fiber-0.2) to 337 MPa (BBL_99_ fiber-0.8) (Fig. 3b). Both *σ*_y_ and *ε*_y_ increased from 185 MPa and 1.7% to 249 MPa and 3.4%. Furthermore, BBL_39_ and BBL_71_ fibers with lower molecular weights exhibit a similar trend, where the tensile strength increases with enhanced shear force during fiber formation. In addition, the mechanical properties of the fibers are strongly dependent on the molecular weight [47]. Under the same shear condition, the tensile strengths of BBL_39_ fiber-0.8 and BBL_71_ fiber-0.8 are 139 ± 8 MPa and 214 ± 13 MPa, respectively (Fig. S27). Fibers spun from high-molecular-weight BBL_165_ (*M*_v_ = 55 kDa; 165 repeating units) featured Young's modulus of 15.3 GPa, *σ*_s_ of 595 MPa and elongation at break of 54%, surpassing commonly used textile fibers [48–50] (Fig. 3b and c). The *σ*_y_ and *ε*_y_ values were 383 MPa and 2.4%, respectively.

The rod-like and ladder-like molecular structure imparts BBL fibers with excellent ultraviolet resistance, chemical stability and thermodynamic stability, significantly broadening their application in the field of intelligent textiles (Fig. 3d, Fig. S28). After 12-h exposure to intense UV irradiation (5000 W m^−2^ at 365 nm, approximately 3500 times the conventional UV light intensity), BBL_99_ fiber-0.8 retained 96% of its tensile strength, similarly preserved after soaking in acetone (99.5 vol%), NaOH (30 wt%) and H_2_SO_4_ (70 vol%) for 24 h, retaining 94%, 91% and 90% of the initial tensile strength, respectively. Owing to their low crystallinity and orientation, BBL_99_ fiber-0.2 and -0.5 retained only 88% and 89% of their tensile strength in NaOH solution, respectively. Similarly, they retained 82% and 85% of their strength in H_2_SO_4_ solution, respectively (Fig. 3e).

The high crystallinity and orientation induced by shear-enhanced liquid-crystal phases confer thermal stability to BBL fibers. Moreover, BBL_99_ fibers are resilient to changes in temperature, as evidenced by a minimal change in tensile strain when heated from −120°C to 250°C while under constant tensile stress (Figs S29 and S30). Figure S31 shows a differential scanning calorimetry (DSC) thermogram for BBL_99_ fiber. As previously reported [36,51], there are no observable thermal transitions between 0°C and 300°C. Thermogravimetric analysis (TGA) indicated mass retention of about 98.2% upon heating to 500°C (Fig. S32a). The fibers exhibited outstanding thermomechanical stability, remaining stable even in liquid nitrogen (Fig. 3f, Fig. S32b–d, Movie S2), which confirms their robust mechanical properties and stability in various environments.

### Semiconductor fiber-based OECTs and fabric arrays

**Figure 4.**
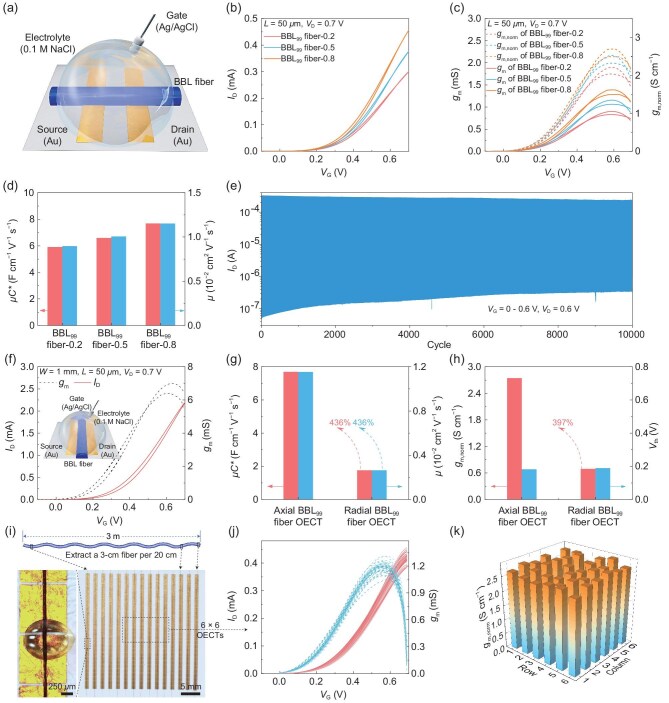
Semiconductor-fiber OECTs. (a) Schematic of an axially oriented BBL-fiber OECT. (b) Transfer characteristics of BBL_99_-fiber OECTs. (c) Transconductance and *g*_m, norm_ of BBL_99_ fiber. (d) μC* and *μ* of BBL_99_ fiber extracted from the transfer curve. (e) Operational stability of a BBL_99_-fiber OECT under periodic square-wave gate bias for almost 25 h. All the devices have identical geometry (*L* = 50 μm). (f) Schematic, transfer curve and transconductance curve of an axially oriented BBL_99_-fiber OECT. (g) Comparison of μC* and *μ* between axially and radially oriented BBL_99_ fiber OECTs. (h) Comparison of *g*_m, norm_ and *V*_th_ for axial and radial BBL_99_-fiber OECTs. All radial devices have the same channel geometry (*L* = 50 μm; *W* = 1 mm; *d* = 5.06 μm). (i) Photograph of a 27 × 15 BBL_99_ fiber-0.8 OECT array fabricated on nanofiber fabrics. The inset shows the device structure (*L* = 50 μm). (j) Transfer and transconductance characteristics of 6 × 6 region from the fabric OECT array at *V*_D_ = 0.7 V. (k) *g*_m, norm_ across the 6 × 6 region of the array.

We evaluated the impact of shear-enhanced liquid-crystal phases on the functionality of BBL_99_ fiber OECTs. The electrical and electrochemical properties of BBL fibers were extracted from OECT transfer and output measurements. The BBL_99_ fiber was positioned across patterned Au source and drain electrodes, with the active channel covered by a 0.1 M NaCl electrolyte and biased via an Ag/AgCl gate electrode (Fig. 4a). All BBL_99_ fiber OECTs show typical n-type accumulation-mode behavior in both output and transfer curves, as well as a standard deviation of the maximum drain current (*I*_ON_) of less than 4.7% for five different devices (Fig. S33). The *I*_ON_/*I*_OFF_ ratio increased from 2.5 × 10^3^ for BBL_99_ fiber-0.2 devices to 6.5 × 10^3^ for BBL_99_ fiber-0.8 OECTs (Fig. 4b and c, Fig. S34). At a drain voltage (*V*_D_) and gate voltage (*V*_G_) of 0.7 V, the drain current (*I*_D_) reaches 0.30 ± 0.01 mA for BBL_99_ fiber-0.2 devices compared to 0.46 ± 0.02 mA for BBL_99_ fiber-0.8 devices. The maximum geometry-normalized transconductance (*g*_m, norm_) increases from 2.21 ± 0.11 S cm^−1^ for BBL_99_ fiber-0.2 devices to 2.76 ± 0.09 S cm^−1^ for BBL_99_ fiber-0.8 devices (Fig. 4c, Table S4). We then calculated the product of charge-carrier mobility and volumetric capacitance (μC*) to be 5.91 ± 0.18, 6.59 ± 0.32 and 7.66 ± 0.48 F cm^−1^ V^−1^ s^−1^ for BBL_99_ fiber-0.2, BBL_99_ fiber-0.5 and BBL_99_ fiber-0.8 devices, respectively (Fig. S34, Table S5). Since C* does not change significantly within the series, it averages 655 to 667 F cm^−3^ (Figs S35–S38), the and *μ* ranges from (8.98 ± 0.65) × 10^−3^ cm^2^ V^−1^ s^−1^ for BBL_99_ fiber-0.2 devices to (1.15 ± 0.08) × 10^−2^ cm^2^ V^−1^ s^−1^ for BBL_99_ fiber-0.8 devices (Fig. 4d).

The enhanced performance of BBL_99_ fiber-based OECTs is attributed to strong π−π interactions and increased crystallinity confirmed with GIWAXS, indicating that the formation of shear-enhanced liquid-crystal phases leads to enhanced charge transport properties of the rigid BBL polymer backbone [52,53]. Figure 4e demonstrates the robustness of the electrical output of a BBL_99_ fiber OECT at *V*_D_ = 0.6 V with sequential gate bias pulses (*V*_G_ = 0–0.6 V) for nearly 25 h. After more than 10 000 switching cycles, the device maintained a stable *I*_ON_/*I*_OFF_ ratio of approximately 10^3^, demonstrating the exceptional electrochemical stability of BBL fibers in aqueous electrolytes. To the best of our knowledge, this represents the highest cycling stability reported among fiber-based OECTs to date. The response time of a BBL_99_ fiber OECT was assessed by exponential fitting, yielding ON/OFF response times of 507 and 44 ms, respectively (Fig. S39). The electrochemical characteristics of previously reported fiber-based OECTs are summarized in Table S6. According to the WAXS data, BBL_99_ fibers exhibit higher orientation than the freestanding BBL_99_ films obtained by spin-coating. As charge transport in organic semiconductors strongly depends on molecular orientation, we evaluated the electrochemical response of BBL_99_ fibers along their axial and radial directions. A BBL_99_ fiber-0.8 was placed in the wet state with its radial direction along the channel between the source and the drain. The electrode and electrolyte setup matched the axial BBL_99_ fiber OECTs, which comprised a fiber with its axis aligned along the channel (Fig. 4f). We then calculated μC*, *μ* and maximum *g*_m, norm_ for radial BBL fiber OECTs to be 1.76 F cm^−1^ V^−1^ s^−1^, 2.64 × 10^−3^ cm^2^ V^−1^ s^−1^ and 0.69 S cm^−1^, respectively (Fig. S40). Benefiting from the high degree of axial alignment, the μC*, *μ* and maximum *g*_m, norm_ extracted from axial fiber OECTs are approximately four times higher compared to those of radial fiber devices (Fig. 4g and h). In axial fiber OECT devices, charge carriers are propagated along highly coherent transport pathways enabled by enhanced π–π stacking due to the strong axial alignment of polymer chains. This ordered molecular orientation facilitates nearly unobstructed carrier transport, leading to a significant enhancement in carrier mobility. In contrast, radial transport in fiber OECTs is hindered by microstructural discontinuities perpendicular to the fiber axis, such as grain misalignments and interfacial boundaries, which introduce additional interfacial scattering and significantly reduce the effective carrier mobility [54]. As a complementary experiment, BBL_99_ fiber-NS was fabricated under near-zero shear conditions. The corresponding OECT devices exhibited higher carrier mobility [(0.623 ± 0.012) × 10⁻^2^ cm^2^ V⁻^1^ s⁻^1^] compared to radial BBL fiber OECTs, thereby further confirming the critical role of molecular chain alignment in promoting efficient charge transport (Fig. S41).

Leveraging the exceptional mechanical and electrochemical properties of BBL fibers, fully textile OECT arrays were fabricated by patterning electrodes onto nanofiber fabrics and arranging BBL fibers in a structured configuration (Fig. 4I, Fig. S42). The transfer characteristics of the 6 × 6 OECT devices in the central region were measured to assess reliability, demonstrating outstanding device uniformity, with an average *g*_m_ of 1.39 ± 0.06 mS and *I*_ON_/*I*_OFF_ ratio of 1557 ± 73 (Fig. 4j and k). These results highlight the process reproducibility of semiconductor-fiber OECTs and their strong compatibility with current textile manufacturing workflows.

### Fabric-level logic circuits

After demonstrating high-performance n-type accumulation-mode fiber OECTs, we fabricated NAND logic circuits with two, three, four and five input signals (Fig. S43). The NAND gate is a fundamental component in digital electronics, and it can be used to build combinational logic circuits such as adders and data selectors [55]. The successful implementation of these circuits highlights the potential of semiconductor fibers for building large-scale 0–1 logic gate circuits, enabling real-time portable logic operations. Poly(benzimidazobenzophenanthroline) fibers, due to their exceptional electrochemical and mechanical properties, are well suited for fabricating fabric logic circuits.

**Figure 5.**
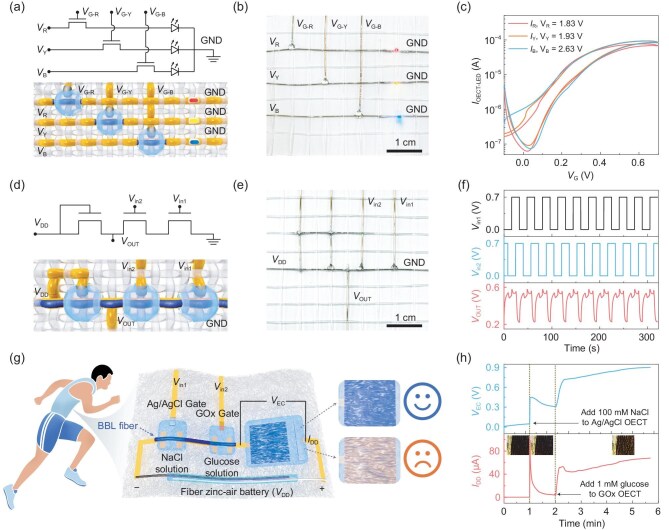
BBL fiber-based logic circuits. (a) Schematic and circuit diagram using fabric BBL-fiber OECTs as switches in series with RYB LEDs. (b) Photograph of the fabric-based OECT-LED circuits. (c) Output characteristics of the fabric circuit showing the positive volt–ampere response. (d) Schematic and circuit diagram of a fabric NAND gate with two input signals. (e) Photograph of the woven NAND gate circuit. (f) Output characteristics of the fabric NAND gate at *V*_DD_ = 0.8 V, where logic ‘0’ and ‘1’ correspond to 0.23 and 0.57 V, respectively. (g) Schematic of the fully fabric-based visualization of a logic sensing wearable system, consisting of a fiber OECT for Na^+^ sensing, a fiber OECT for glucose sensing, a fabric-based electrochromic device (EC) and a fiber Zn–air battery energy storage device. (h) Visualization performance of the system in response to ionic and glucose stimuli. All devices were fabricated using BBL_99_ fiber-0.8.

To integrate signal conversion and digital logic operations into smart textiles, we used weaving technology to construct fabric-level logic circuits with semiconductor fibers. BBL_99_ fiber-0.8 was combined with polyimide fibers bearing patterned electrodes to form the source, drain and channel regions. Conductive Ag/AgCl-coated nylon fibers served as gate electrodes. These functional fibers were woven into fabric, and solid electrolytes were applied at fabric joints to create fabric-level OECTs. The primary function of a transistor device is to serve as an electronic switch in a circuit. The fabric BBL OECT was connected in series with light-emitting diode (LED) drivers and integrated into the fabric (Fig. 5a and b). As the *V*_G_ increased, circuit current and red, yellow, blue (RYB) LED brightness increased accordingly, reaching a fully conductive state at *V*_G_ = 0.6 V (Fig. 5c). Fabric NAND gate logic circuits were also fabricated using BBL-fiber OECTs (Fig. 5d and e). These fabric NAND gate circuits showed excellent performance comparable to planar devices and can communicate 0–1 digital signals (Fig. 5f). The ON-to-OFF output transitions (from digital ‘1’ to ‘0’) confirm the correct logic functionality. This capability enables the development of more complex devices and circuits, highlighting the potential for integrating intelligent circuits into textiles.

Owing to the logical versatility of NAND circuits, their application can be extended to non-invasive multi-marker sensing of biochemical indicators in human sweat. The high biocompatibility of fabric-based sensing platforms supports continuous monitoring of chronic conditions such as diabetes (Fig. 5g). In this system, the gate of a nanofiber-based OECT is functionalized with glucose oxidase (GOx) for selective glucose detection in sweat. Another OECT device with an Ag/AgCl gate responds to Na^+^ and K^+^ ions. Integration of these two OECTs forms a NAND logic-based multi-marker sensing unit. Replacing the resistive OECT in the NAND circuit with a fabric-based electrochromic (EC) device (Figs S44 and S45), integrated with fiber zinc–air batteries and functionalized OECTs, enables the creation of a fully textile-based, visually responsive wearable logic sensing system. When both OECTs are off, the driving voltage (*V*_EC_) remains low, and the EC device appears dark brown. Activation of the Ag/AgCl OECT by sweat simulant exposure slightly increases *V*_EC_, but it remains below the threshold for EC color change. At this stage, the EC device remains dark brown. When the GOx OECT interacts with glucose-containing sweat simulant, the enzymatic oxidation of glucose releases electrons, increases *V*_G_ and alters *I*_D_, raising *V*_EC_ to approximately 0.9 V [56]. This voltage triggers a color transition in the EC device from dark brown to dark gold, visually indicating physiological state (Fig. 5h). This fully textile-integrated sensing platform meets the requirements for non-invasive, wearable and visually responsive real-time health monitoring.

## CONCLUSION

In summary, we demonstrate that shear-induced formation of a lyotropic LC phase enabled the fabrication of BBL fibers with pronounced uniaxial alignment. This molecular ordering markedly enhances both mechanical and electrochemical performance, yielding anisotropic charge transport, where axial carrier mobility and transconductance were approximately four times higher compared to the radial direction. The fibers exhibit a yield strength of 383 MPa, tensile strength of 600 MPa and elongation at break of 54%, alongside excellent environmental stability. These properties make them well suited for scalable textile manufacturing and high-performance wearable electronics. Furthermore, the development of a fully textile-integrated visual logic sensing platform integrating fiber-based sensing, energy storage and logic elements paves the way for real-time health monitoring and precision medical applications.

## METHODS

The synthetic strategies and experimental parts of this work are presented in the supplementary data. These methods include the fabrication of fibers, fiber OECTs, fabric circuits and the associated electrochemical and mechanical property characterization. More detailed information is provided in the online Supplementary data.

## Supplementary Material

nwaf331_Supplemental_Files
