# Supplementary material for: Shear-enhanced liquid-crystal spinning of conjugated polymer fibers
Source: Natl Sci Rev. 2025 Aug 13;12(10):nwaf331. doi: 10.1093/nsr/nwaf331 (PMC12485984; doi:10.1093/nsr/nwaf331)
Supplement: nwaf331_Supplemental_Files [file nwaf331_supplemental_files.zip › Supplementary data-r.pdf]

Supplementary data

Shear-Enhanced Liquid Crystal Spinning of Conjugated Polymer Fibers

Hao Jiang[1,†], Chi-Yuan Yang[2,†], Deyu Tu[2], Yueheng Zhong[1], Zhu Chen[3], Wei Huang[4], Liang-Wen Feng[3], Hengda Sun[1], Christian Müller[5], Antonio Facchetti[6], Hongzhi Wang[1,*], Simone Fabiano[1,2,*] and Gang Wang[1,7,*]

[1]State Key Laboratory of Advanced Fiber Materials, College of Materials Science and Engineering, Donghua University, Shanghai 201620, China;

[2]Laboratory of Organic Electronics, Department of Science and Technology, Linköping University, Norrköing SE-60174, Sweden;

[3]Key Laboratory of Green Chemistry & Technology, Ministry of Education, College of Chemistry, Sichuan University, Chengdu 610065, China;

[4]School of Automation Engineering, University of Electronic Science and Technology of China, Chengdu 611731, China;

[5]Department of Chemistry and Chemical Engineering, Chalmers University of Technology, Göteborg 41296, Sweden;

[6]School of Materials Science and Engineering, Georgia Institute of Technology, Atlanta, Georgia 30332, USA;

[7]Yuyue Home Textile Co., Ltd, Binzhou 256623, China

***Corresponding authors.** E-mails: wanghz@dhu.edu.cn; simone.fabiano@liu.se; gwf8707@dhu.edu.cn

† Equally contributed to this work.

Supporting Information Text

Materials

BBL was synthesized based on previous studies [1] with viscosity-average molecular weights (*M*v) of 13 kDa, 17.9 kDa, 23.6 kDa, 32 kDa, and 55 kDa, named BBL_{39}, BBL_{54}, BBL_{71}, BBL_{99}, and BBL_{165}. BBB was synthesized based on previous studies [2]. Methanesulfonic acid (MSA), NaOH (97%), and ethanol were purchased from Aladdin. Poly (sodium 4-styrenesulfonate) (average *M*w ≈ 1000 kDa, powder) was obtained from Sigma Aldrich. Concentrated sulfuric acid (≈98%), glycerol (≥99.0%), D-sorbitol (≥99.5%), NaCl (≥99.5%), and acetone (AR, ≥99.5%) were obtained from China National Pharmaceutical Group Corporation. Unless otherwise stated, all materials were used as received.

Methods

Semiconductor fiber preparation. The BBL_{99} fiber was fabricated through a liquid crystal wet spinning method. BBL_{99} was dissolved in MSA (5 mg mL^{-1}) under high-speed stirring. The BBL_{99}-MSA spinning solution was pumped into the deionized water-filled coagulation bath at 20 °C with a rate of 0.2, 0.5, and 0.8 mL min^{-1} using a syringe thruster (KD Scientific, Legato 101) and spinnerets with different inner diameters. During the solvent exchange, dark-purple BBL fibers were obtained. The coagulated BBL fibers were drawn twice sequentially at a draw ratio of 1:1.08. Finally, the fibers were continuously collected on fiber spools for drying and then annealed at 200 °C. Except that the concentration of the BBL_{39}-MSA solution is 10.1 mg mL^{-1}, the preparation method for BBL_{39} fibers is the same as for BBL_{99} fibers. PBFDO-DMSO solution (10 ~ 12 mg mL^{-1}, VOLT-AMP Optoelectronics Tech. Co.) was diluted to 7.6 mg mL^{-1} as the spinning solution, with H_2SO_4-H_2O solution used as the coagulation bath, spun with the same parameters as BBL_{99} fibers, and dried at room temperature to obtain PBFDO fibers. PEDOT:PSS aqueous solution (1.1 ~ 1.3% solid content, Clevios™ PH1000, Heraeus Electronic Materials) was freeze-dried to solid PEDOT:PSS and then configured to 6.8 mg mL^{-1} aqueous solution. PEDOT:PSS fibers were obtained using H_2SO_4 solution as the coagulation bath and spinning with the same parameters as BBL_{99} fibers. The fibers were dried at room temperature. BBL_{99} fiber-NS was fabricated under near-zero shear (NS)

conditions.

Fluid Simulation and Finite Element Analysis. In this study, numerical simulations of fluid flow were conducted based on the finite element method (FEM). The pressure-coupled SIMPLE (PC-SIMPLE) algorithm, implemented within a C++-based finite element framework, was adopted to solve the incompressible Navier-Stokes equations and effectively handle the pressure-velocity coupling challenge. The computational domain was divided into mesh elements optimized for finite element analysis, with particular emphasis placed on mesh refinement within regions of high gradients to improve numerical accuracy. The turbulence characteristics were simulated employing the standard k–ε turbulence model, wherein turbulent viscosity was estimated by solving the transport equations for turbulent kinetic energy (k) and turbulence dissipation rate (ε). Boundary conditions were defined as follows: a velocity-inlet condition at the inlet, an outflow boundary at the outlet, and stationary walls obeying no-slip boundary conditions. The flow field was initialized via the hybrid initialization method, followed by 200 iterative computations to ensure convergence. Specifically, residuals for momentum and fluid volume fraction were maintained below 10^{-5}, and those for the enthalpy conservation equation were reduced to below 10^{-7}, thus satisfying rigorous convergence criteria.

Molecular Dynamics (MD). All molecular dynamics simulations were conducted using Materials Studio v6.0.0 (Accelrys Software Inc., San Diego, CA, 2011), employing the Discover module for simulation setup. The COMPASS force field was used throughout, which has been previously validated for π–π stacking interactions and charge transport behavior in conjugated polymer systems[3]. Electrostatic interactions were calculated using the Ewald summation method. The simulation cell contained 10 BBL polymer chains, each comprising 30 repeating units, randomly dispersed in explicit methanesulfonic acid (MSA) solvent molecules at a BBL:MSA molar ratio of 1:100. The initial configurations were generated through random placement, followed by energy minimization and a 100 ps equilibration under the NPT ensemble at 298 K. Temperature was controlled using a Nosé thermostat, and pressure regulation was performed using the Berendsen barostat. Subsequently, an annealing process consisting of 10 heating–cooling cycles between 300 K and 500 K under NPT conditions was carried out to eliminate any artificial ordering effects. Annealing was followed by a production MD simulation lasting 100 ps at 298 K

and 0.1 GPa, with a time step of 0.5 fs. To investigate the effects of shear, a linear velocity gradient along the y-axis (shear direction) was applied (simple shear) while maintaining periodic boundary conditions. Three shear rates (0.0042 ps^{-1}, 0.0105 ps^{-1}, and 0.0169 ps^{-1}) were applied to map the experimentally relevant flow conditions. Structural analyses were performed on the characteristic nitrogen (N) atoms of the BBL molecules. Specifically, mean square displacement (MSD), radial distribution function (RDF) with a 4 Å cutoff, and radius of gyration (Rg) were calculated to assess molecular diffusion, aggregation, and chain extension, respectively.

Polarized optical microscopy and *in situ* observation. Polarized optical microscopy (POM) images of the BBL fibers were obtained with an Olympus BX60 optical microscope. The fluid flow in the microchannel is driven and precisely controlled by using a syringe pump system (KD Scientific, Legato 101). BBL_{99}-MSA was configured as experimental fluid at a concentration of 5 mg mL^{-1}. Glass capillaries with inner diameters of 0.2 mm were used as microfluidic channels for *in situ* polarization analysis to characterize the fluid state during the spinning process.

Critical concentration of BBL. According to Flory's molecular theory [4], the axial ratio (x) of a rigid-rod molecule can be quantitatively estimated using the following formula:

$$x = \left(\frac{M}{M_{\mathrm{u}}}\right)\left(\frac{l_{\mathrm{u}}}{d}\right)$$

Where M represents the molecular weight, M_{u} denotes the molecular weight of the repeat unit (334), l_{u} corresponds to the length of the repeating unit (about 1.25 nm) [5], and d refers to the average chain diameter (1.2 nm) [6]. The critical concentration (φ^*) can be calculated using the axial ratio through the following formula:

$$\varphi^* = \frac{8}{x}\left(1 - \frac{2}{x}\right)$$

where φ^* is the critical concentration in volume fraction. Ultimately, an equation relating critical concentration in terms of mass fraction to molecular weight is derived:

$$c^*(M) \approx \frac{8\rho_p M_u d}{10^3 l_u}\frac{1}{M} - \frac{16\rho_p M_u^2 d^2}{10^6 l_u}\frac{1}{M^2}$$

where ρ_p represents the density of the solute.

X-ray Scattering. BBL_{99}-fiber: Grazing-incidence wide-angle X-ray Scattering (GIWAXS) experiments were performed at Xenocs Xeuss 2.0. The X-ray energy was 8.73 keV, and the incidence angle was 0.12°. The total exposure time was 1800 s. The scattered X-rays were recorded by a charge-coupled device detector located 210.783 mm from the sample. BBL_{39}-fiber: GIWAXS experiments were performed at the beamline BL02U2 system at Shanghai Synchrotron Radiation Facility (SSRF), China. The X-ray energy was 9.5 keV, and the incidence angle was 0.12°. The total exposure time was 10 s. The scattered X-rays were recorded by a charge-coupled device detector located 207.456 mm from the sample.

Wide-angle X-ray scattering (WAXS) experiments were performed using a Rigaku Rapid II diffractometer, and the wavelength of the X-ray source was 0.154 nm. The axial direction of BBL fibers is perpendicular to the incident X-ray beam and parallel to the meridian of the two-dimensional WAXS pattern. The BBL fibers and film samples were exposed for 15 s and 60 s at ambient conditions. The scattering images were captured with a Hypix-6000 detector; the sample-to-detector distance was 70 mm.

Small-angle X-ray scattering (SAXS) measurements were undertaken on the beamline BL19U2 SAXS system at SSRF, China. The radiation energy of the X-rays used was 12.4 keV at a wavelength of 1.03 Å. The distance between the detector and the fibers is 3180 mm. The BBL fibers were placed in neatly oriented bundles, anchored parallel to a suitable sample stage with an aperture for X-ray irradiation. Samples were exposed to X-rays for 3 s to obtain 2D SAXS patterns of the BBL fibers.

Morphology and mechanical properties of fibers. The surface and cross-sectional scanning electron microscopy (SEM) images of fibers were obtained by FESEM (SU-8010, Hitachi, Japan). The mechanical properties of the fibers were investigated with the Instron universal material testing system (INSTRON 5969, USA). The stretch rate was 100 mm min^{-1}.

Thermomechanical properties of fibers. Thermomechanical properties were characterized using a DMA Q800 in constant-force mode from TA Instruments. The fiber was held under a controlled force (15 MPa), and the strain was monitored during a

temperature ramp (5 K min^{-1}) from −140 to 300 °C. Thermogravimetric analysis (TGA) was carried out using a TGA/DSC 3+ instrument from Mettler Toledo and a DSC 214 Polyma instrument from Netzsch. The mass loss and heat flow of solid materials were obtained in nitrogen at a heating rate of 10 K min^{-1}.

Environmental stability of fibers. Ultraviolet (UV): The BBL_{99} fibers were arranged in parallel on a glass slide and irradiated with a 365 nm UV LED lamp at a power of 5000 W m^{-2} (about 3500 times the conventional UV light intensity) for 12 hours, and then the tensile test was performed. BBL fibers were handled in a constant-temperature humidifier sample chamber at 25 °C.

Chemical resistance: A quantity of the fibers was positioned in acetone (99.5 vol%), H_2SO_4 (70 vol%), and NaOH (30 wt%) solutions. After 24 h, the processed fibers were cleaned with deionized water and dried naturally at room temperature for tensile characterization.

Fiber OECTs and NAND logic circuit fabrication and characterization. The metals employed for the electrodes were 3 nm Cr and 30 nm Au. The width of the transistor channel was 50 μm, and the length depended on the fiber diameter. 0.1 mol L^{-1} NaCl aqueous solution was applied as the electrolyte, and an Ag/AgCl pellet electrode was employed as the gate electrode.

Electrochemical impedance spectroscopy (EIS) characterizations were carried out on the Princeton Electrochemical Workstation (PMC CHS08A) at room temperature. For EIS characterizations, 0.1 mol L^{-1} NaCl aqueous solution was applied as the electrolyte, a platinum mesh (2.5 × 2.5 cm) served as the counter electrode, and Ag/AgCl (saturated) was employed as the reference electrode. For volumetric capacitance characterization, The working electrode was set to −0.7 V, and spectra were measured at 10^{-1} Hz–10^{2} Hz. The results were fitted using the equivalent circuit model $Rs(Rp||Q)$.

OECTs and inverters were performed utilizing a Keithley 4200 semiconductor parameter analyzer. The continuous gate pulses were employed for the stability measurements through a Tektronix 3390 wave function generator.

Fabric-level logic circuit. Gel electrolyte: The PSSNa, glycerol, D-sorbitol, and deionized water (weight ratio of 5: 2: 2: 1) were mixed through a sonication and stirring process

(50 °C). The prepared gel electrolyte had good shape retention and excellent electrochemical performance.

The patterned electrodes on polyimide fibers were prepared by mask evaporation, and the BBL composite fibers were prepared by laminating BBL fibers on the electrodes. The BBL composite fibers and fibers coated with Ag/AgCl electrodes and gel electrolytes were woven into the fabric. Dripping gel electrolytes were used to prepare the channel of fabric logic OECTs.

Textile OECT array and visual logic sensing. The patterning of fabric electrodes is achieved using a mask deposition process (10 nm Cr and 100 nm Au) on the nanofiber fabric substrate prepared by electrospinning. Subsequently, by orderly aligning BBL semiconductor fibers on these electrodes, combined with gel electrolytes and independent Ag/AgCl gates, fabric organic electrochemical transistor (OECT) arrays can be constructed.

Based on preparation strategies similar to the aforementioned OECT arrays, OECT units with specific functionalities can be further developed on the same textile substrate. By preparing Ag/AgCl-OECT and GOx (Glucose Oxidase)-modified OECT on nanofiber fabrics, a multifunctional response capability to markers such as ions (e.g., Na^+, K^+) and glucose can be realized. By spraying an isopropanol suspension of BBL on a fabric electrode with an area of approximately 8 × 8 mm, an electrochromic (EC) working electrode is constructed, with a drop-cast Ag/AgCl electrode as the EC counter electrode and 0.1 M NaCl solution as the EC electrolyte. Finally, the fabric OECT, EC device, and fiber-based zinc-air battery are integrated to form a fully textile-based wearable logic sensing system.

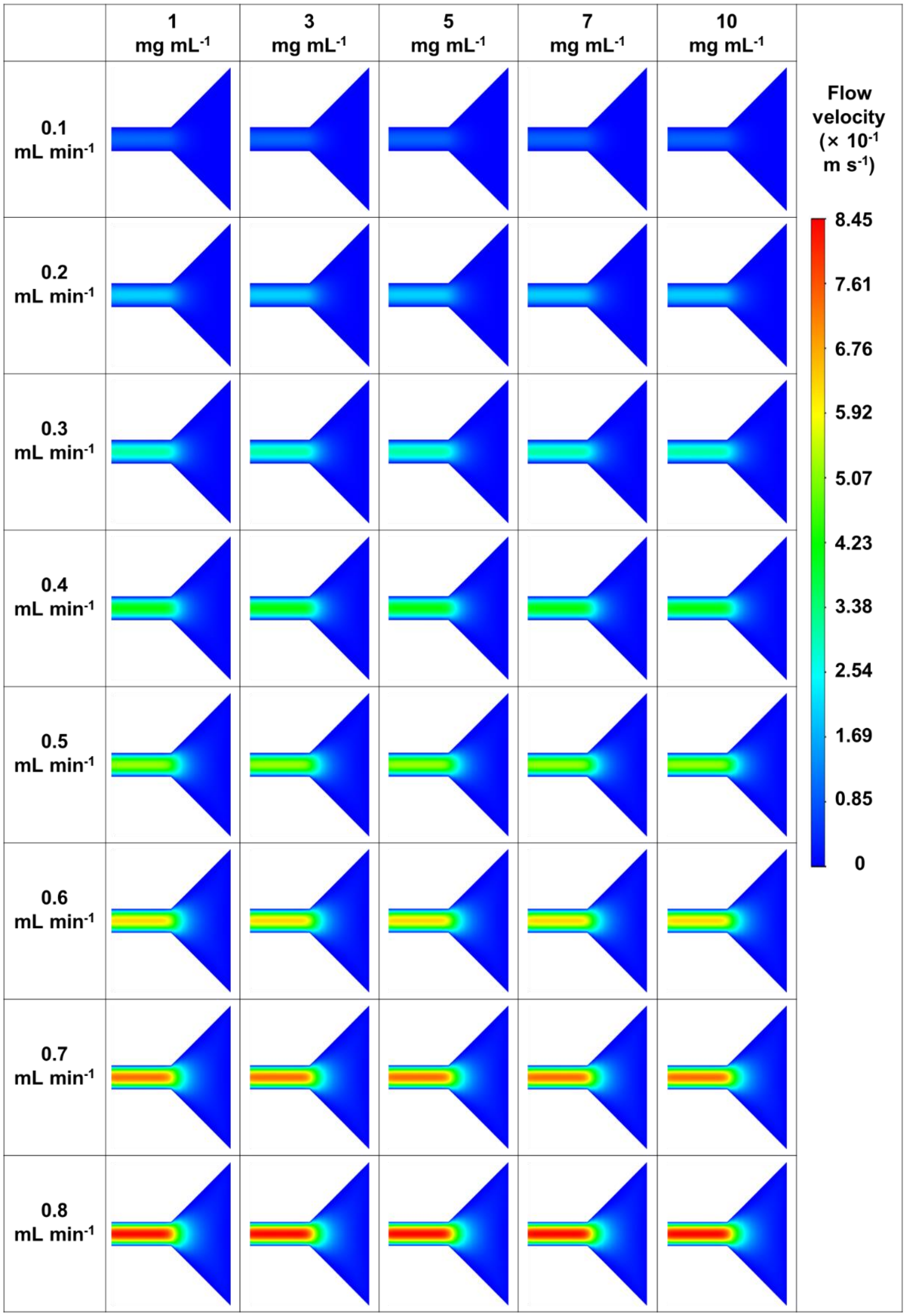

Figure S1 Finite element simulation of the flow velocity distribution.

Finite element simulation of the flow velocity distribution in a 0.2 mm diameter microfluidic channel for different concentrations (1, 3, 5, 7, and 10 mg mL^{-1}) and different spinning speeds (0.1 ~ 0.8 mL min^{-1}) of BBL$_{99}$-MSA solution.

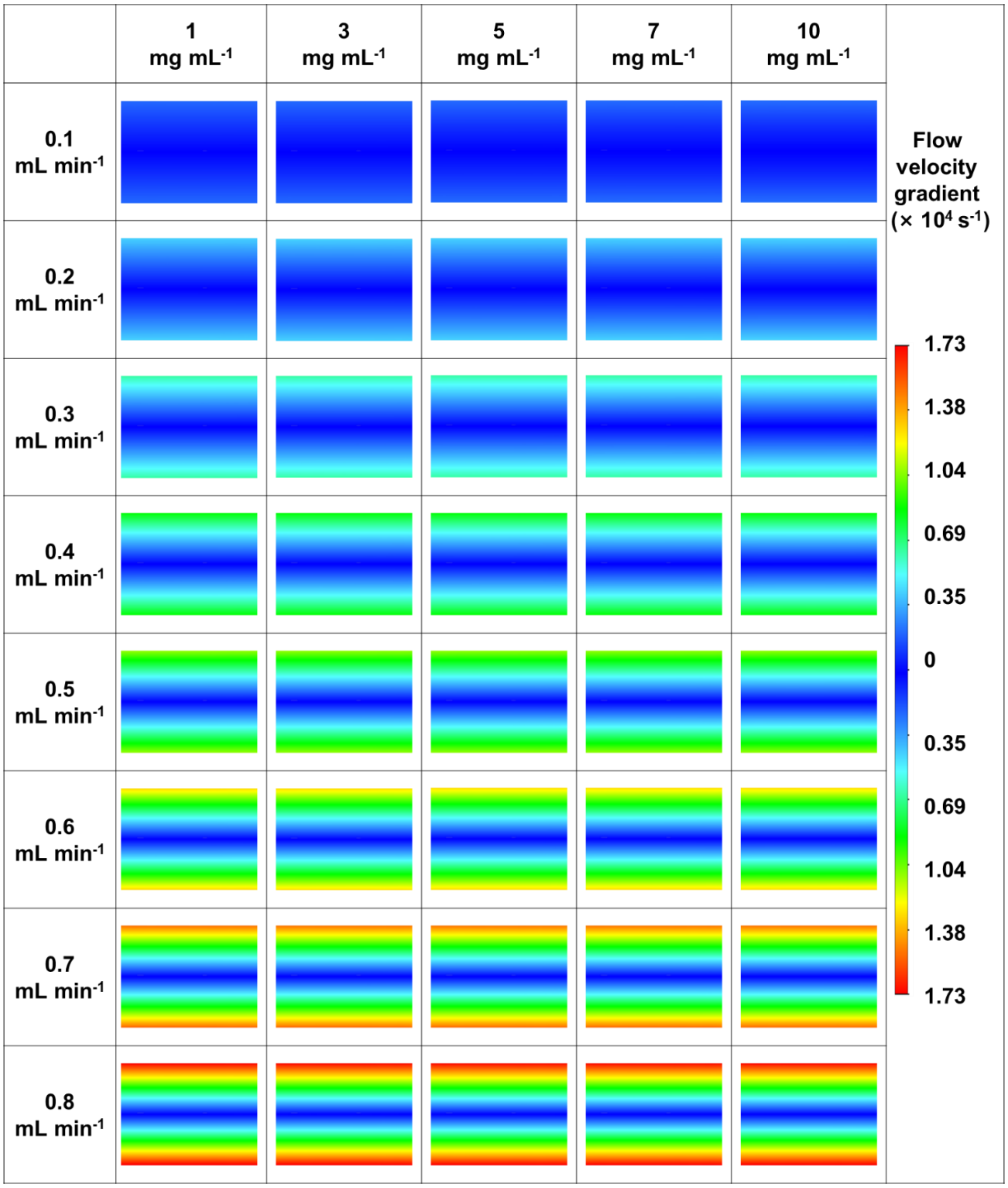

Figure S2 Finite element simulation of the flow velocity gradient distribution.

Finite element simulation of the flow velocity gradient distribution in a 0.2 mm diameter microfluidic channel for different concentrations (1, 3, 5, 7, and 10 mg mL^{-1}) and different spinning speeds (0.1 ~ 0.8 mL min^{-1}) of BBL$_{99}$-MSA solution.

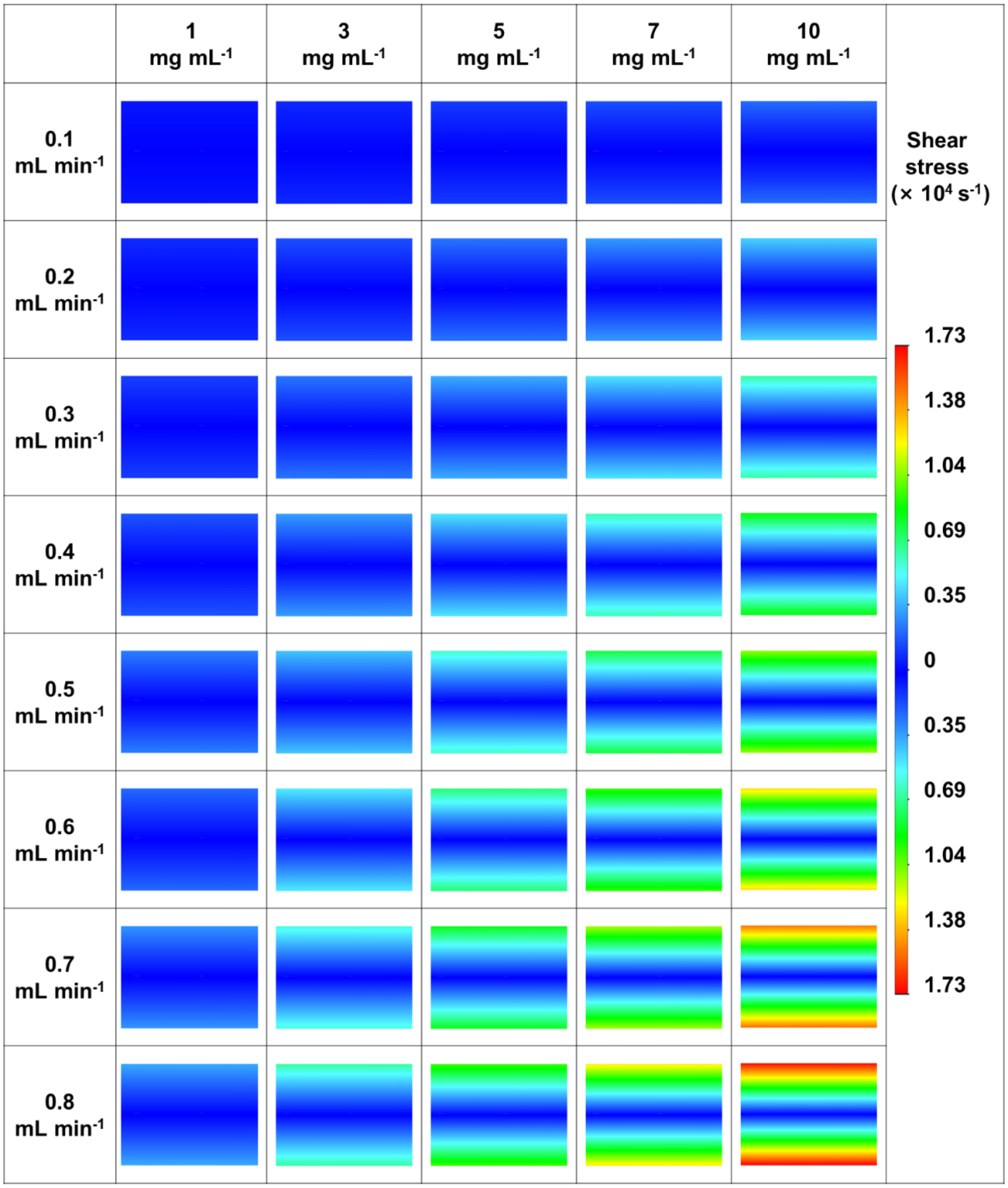

Figure S3 Finite element simulation of the shear stress distribution.

Finite element simulation of the shear stress distribution in a 0.2 mm diameter microfluidic channel for different concentrations (1, 3, 5, 7, and 10 mg mL^{-1}) and different spinning speeds (0.1 ~ 0.8 mL min^{-1}) of BBL$_{99}$-MSA solution.

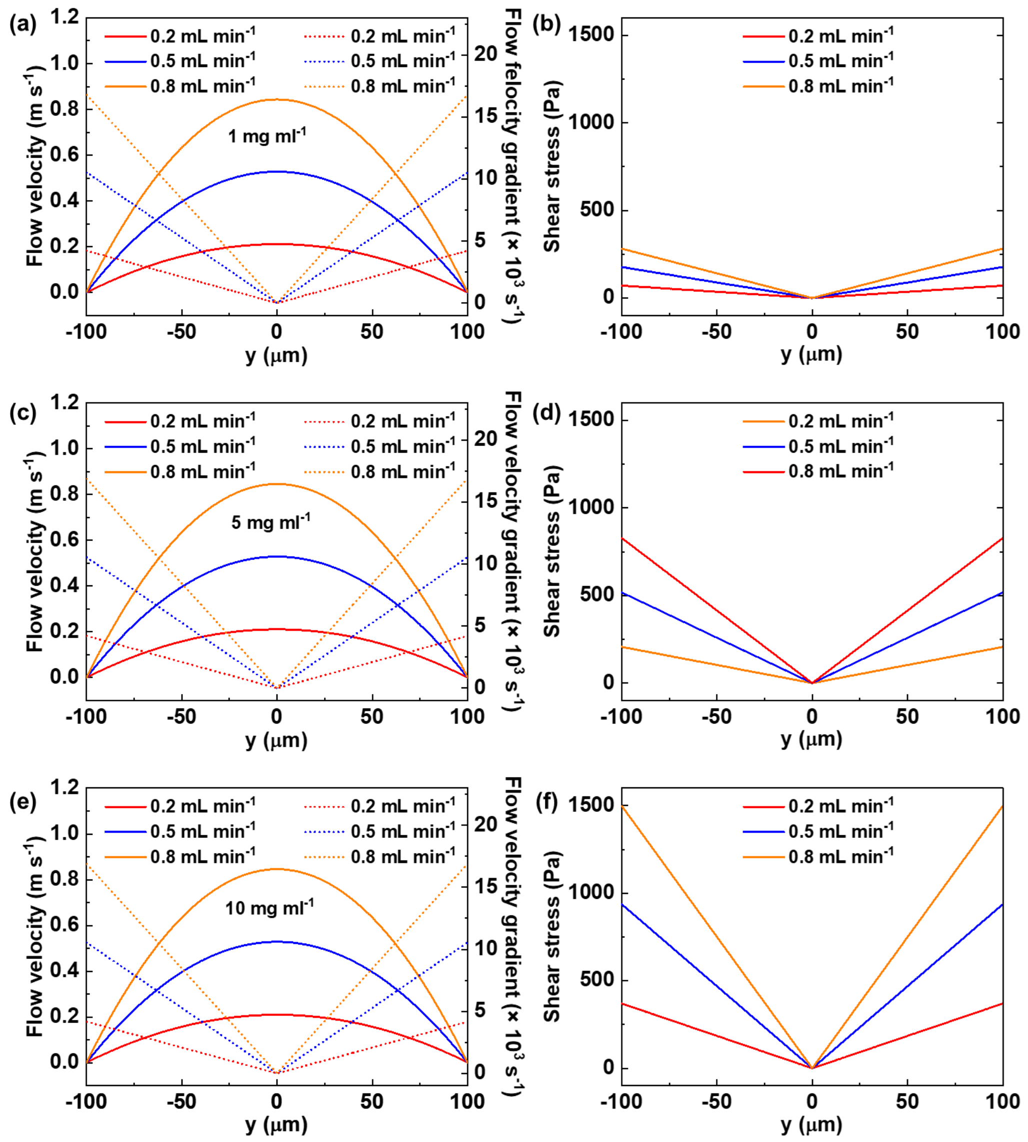

Figure S4 The flow velocity, flow velocity gradient, and shear stress of BBL99-MSA solution.

The flow velocity, flow velocity gradient, and shear stress in a 0.2 mm diameter microfluidic channel for different concentrations (1, 3, 5, 7, and 10 mg mL^{-1}) and different spinning speeds (0.1 ~ 0.8 mL min^{-1}) of BBL99-MSA solution.

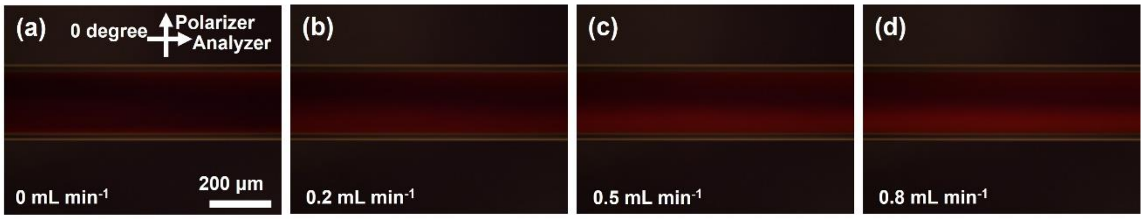

Figure S5 POM photographs of the BBL_{99}-MSA solutions.

POM photographs of the BBL_{99}-MSA solutions in a 0.2 mm diameter glass tube in pressure-driven nematic flows at 0° to the analyzer direction with 0 (a), 0.2 (b), 0.5 (c), 0.8 (d) mL min^{-1}.

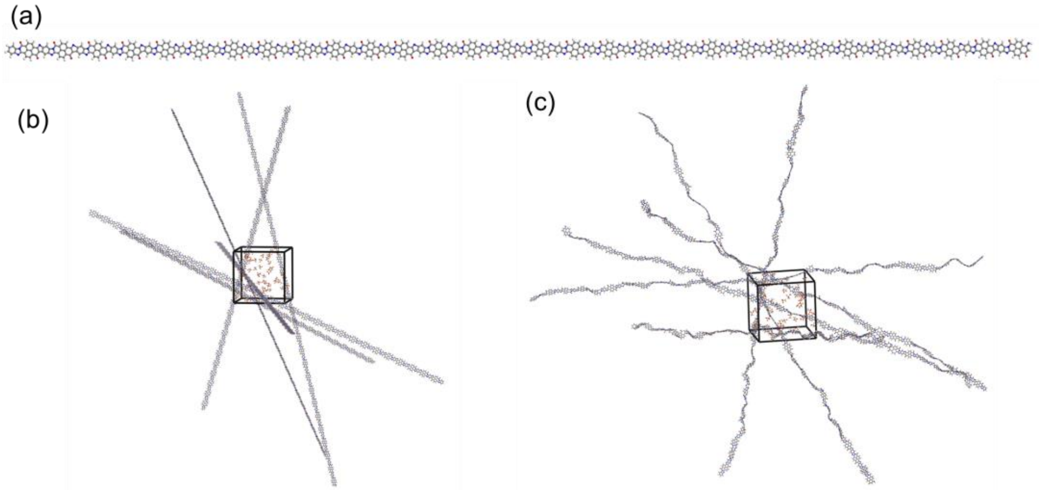

Figure S6 Molecular Dynamics Model of the BBL-MSA System.

(a) BBL chain (united by 30 repeating units). (b) A single simulation unit before annealing. (c) A single simulation unit after annealing

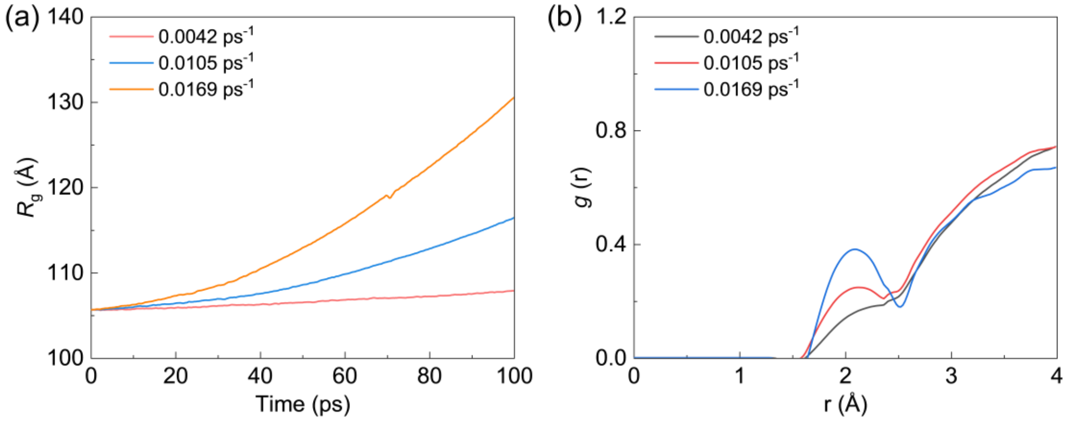

Figure S7 Radius of gyration (a) and radial distribution (b) function of the N atoms.

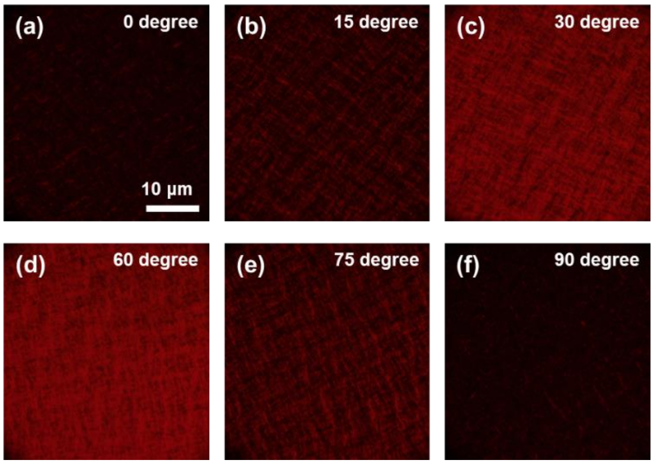

Figure S8 POM photographs of the 90 mg mL^{-1} BBL_{99}-MSA solutions.

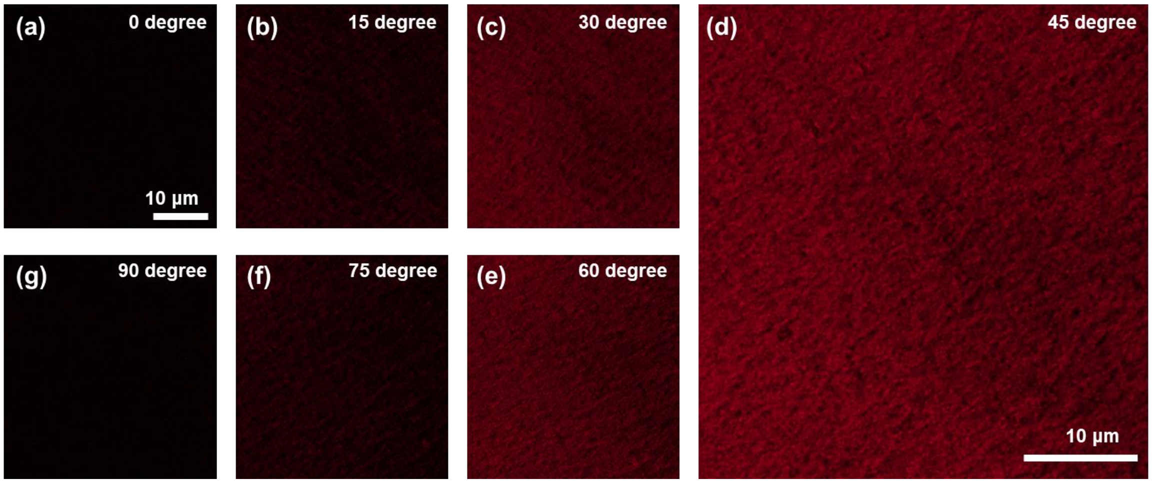

Figure S9 POM photographs of the 123.73 mg mL^{-1} BBL_{71} (where 71 indicates the average number of repeating units; viscosity-average molecular weight (M_v) = 23.6 kDa)-MSA solutions at 0°, 15°, 30°, 45°, 60°, 75°, and 90° to the analyzer direction.

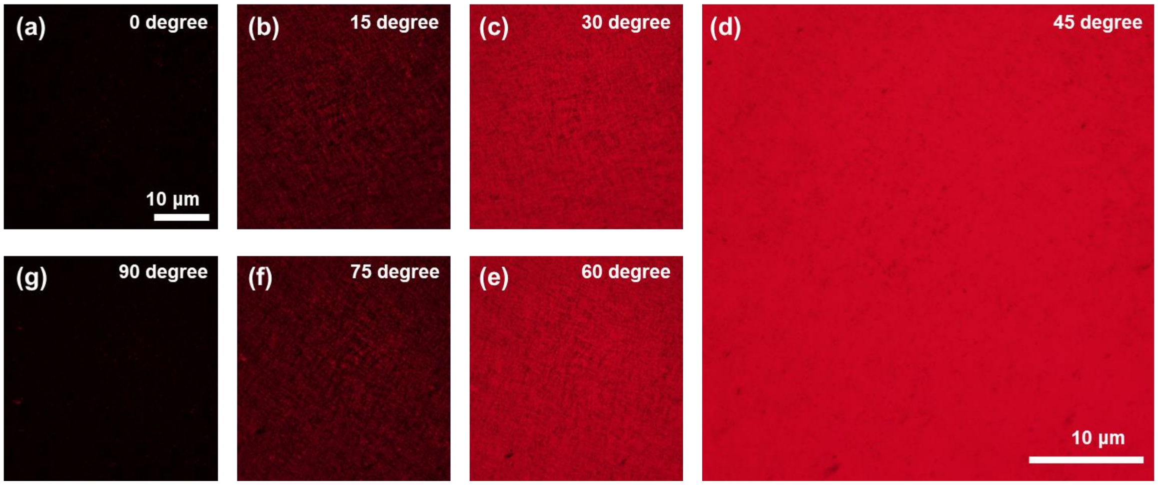

Figure S10 POM photographs of the 165 mg mL^{-1} BBL_{54} (where 54 indicates the average number of repeating units; viscosity-average molecular weight (M_v) = 17.9 kDa)-MSA solutions at 0°, 15°, 30°, 45°, 60°, 75°, and 90° to the analyzer direction.

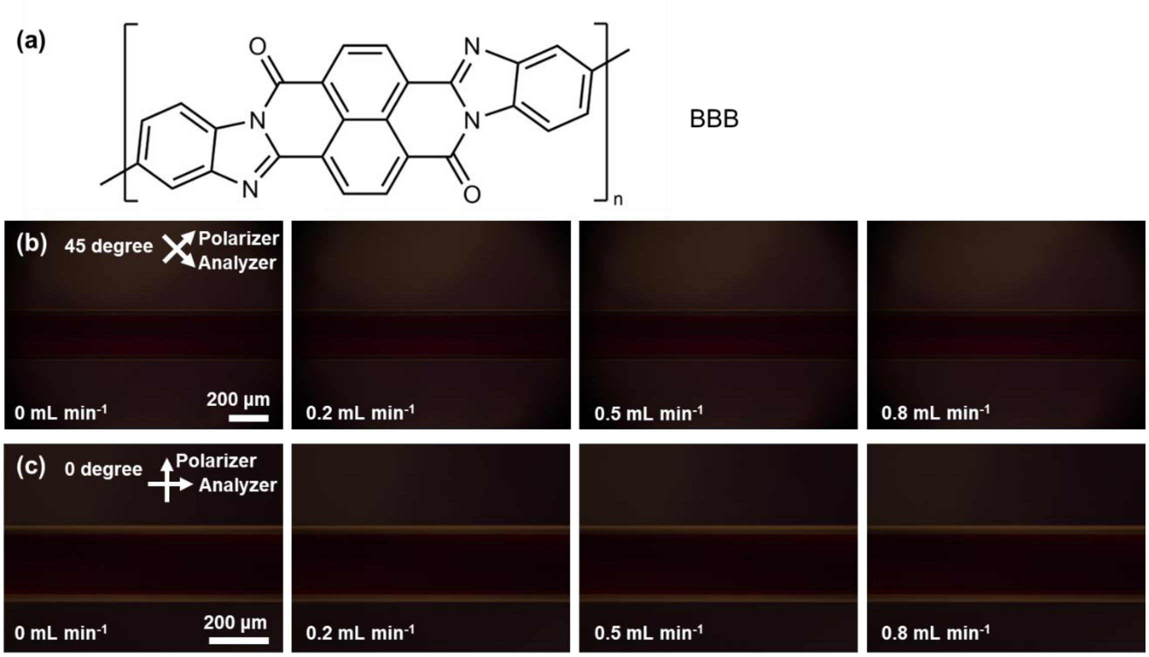

Figure S11 POM photographs of the BBB-MSA.

(a) Chemical structure of BBB. (b-c) POM images of the BBB-MSA solutions in a 0.2 mm diameter glass tube in pressure-driven nematic flows at 45° (b) and 0° (c) to the analyzer direction with 0, 0.2, 0.5, and 0.8 mL min^{-1}.

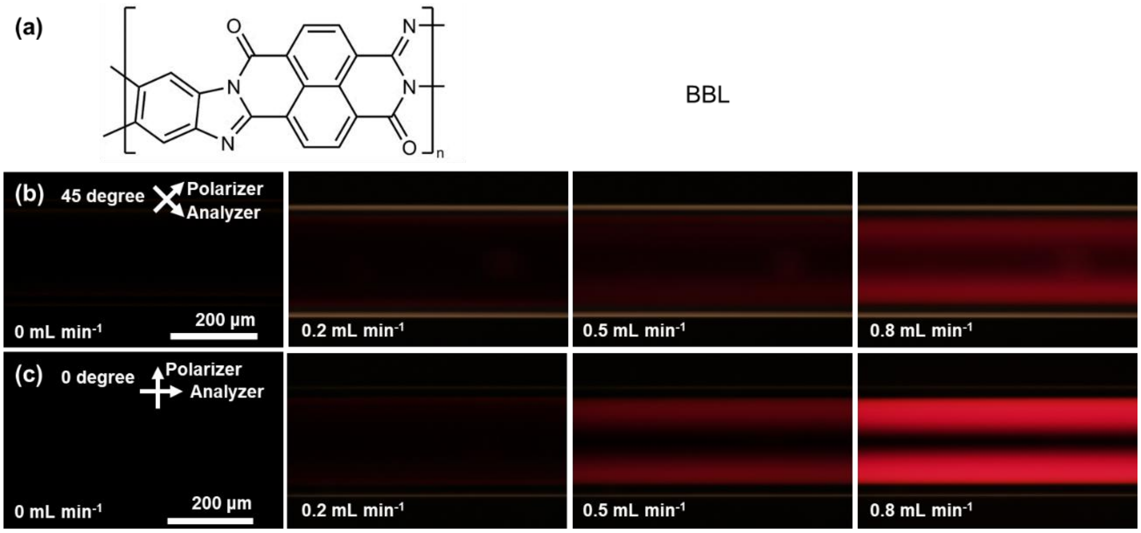

Figure S12 POM images of the BBL_{39}-MSA solutions

(a) Chemical structure of BBL. (b-c) POM images of the BBL_{39}-MSA solutions in a 0.2 mm diameter glass tube in pressure-driven nematic flows at 45° (b) and 0° (c) to the analyzer direction with 0, 0.2, 0.5, and 0.8 mL min^{-1}.

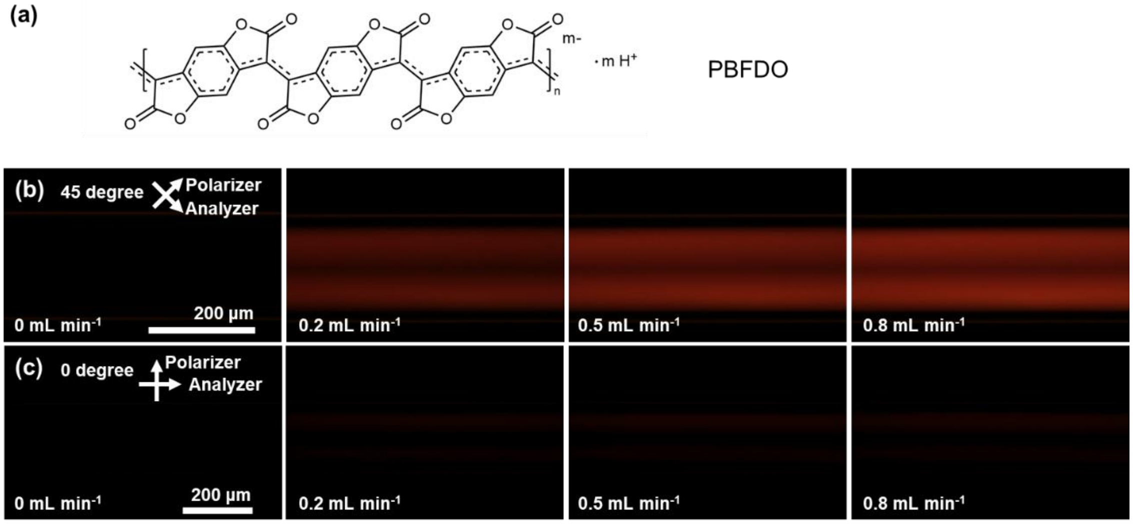

Figure S13 POM images of the PBFDO-DMSO solutions.

(a) Chemical structure of PBFDO. (b-c) POM images of the PBFDO-DMSO solutions in a 0.2 mm diameter glass tube in pressure-driven nematic flows at 45° (b) and 0° (c) to the analyzer direction with 0, 0.2, 0.5, and 0.8 mL min^{-1}.

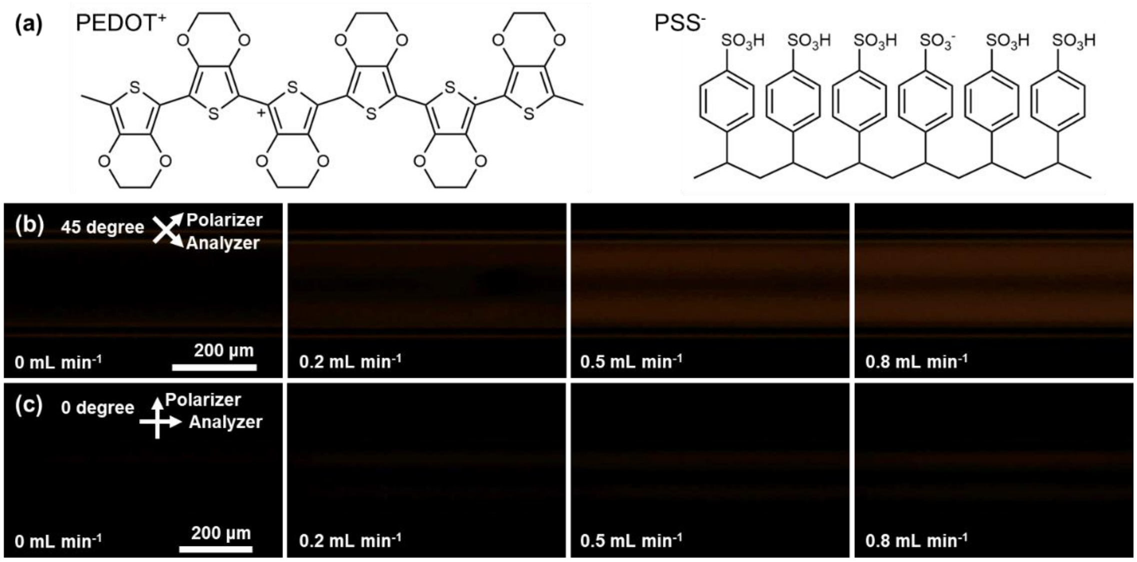

Figure S14 POM images of the PEDOT:PSS-H_2O solutions.

(a) Chemical structure of PEDOT:PSS. (b-c) POM images of the PEDOT:PSS-H_2O solutions in a 0.2 mm diameter glass tube in pressure-driven nematic flows at 45° (b) and 0° (c) to the analyzer direction with 0, 0.2, 0.5, 0.8 mL min^{-1}.

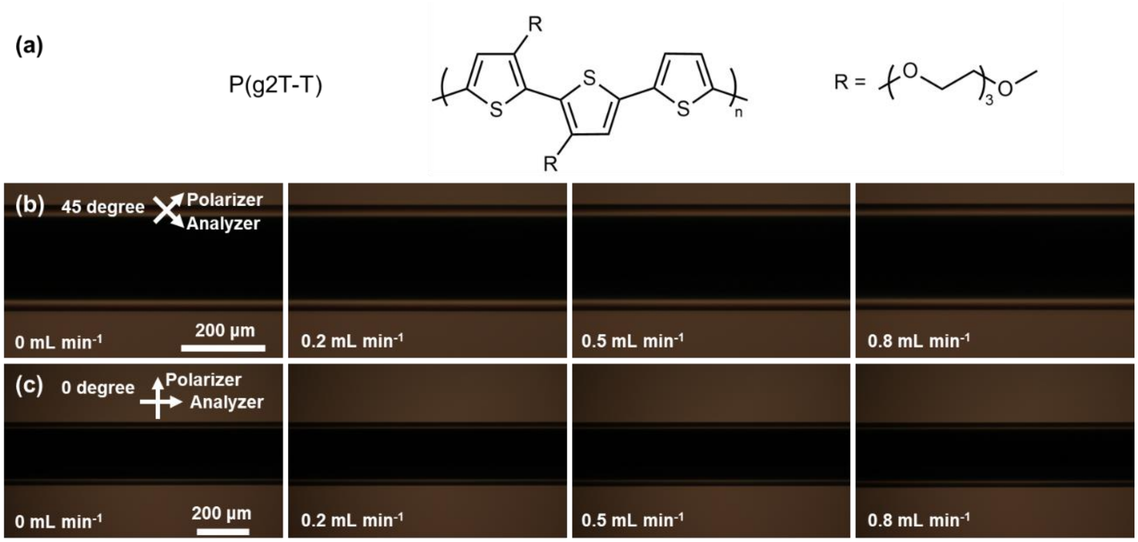

Figure S15 POM images of the p(g2T-T)-MSA solutions.

(a) Chemical structure of p(g2T-T). (b-c) POM images of the p(g2T-T)-MSA solutions in a 0.2 mm diameter glass tube in pressure-driven nematic flows at 45° (b) and 0° (c) to the analyzer direction with 0, 0.2, 0.5, 0.8 mL min^{-1}.

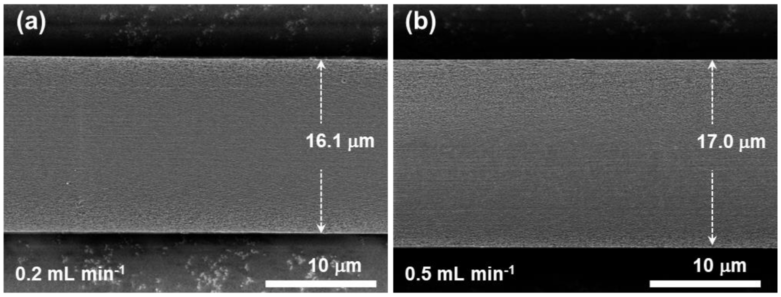

Figure S16 SEM images of the BBL fiber.

SEM images of the BBL fiber-0.2 (a) and BBL fiber-0.5 (b).

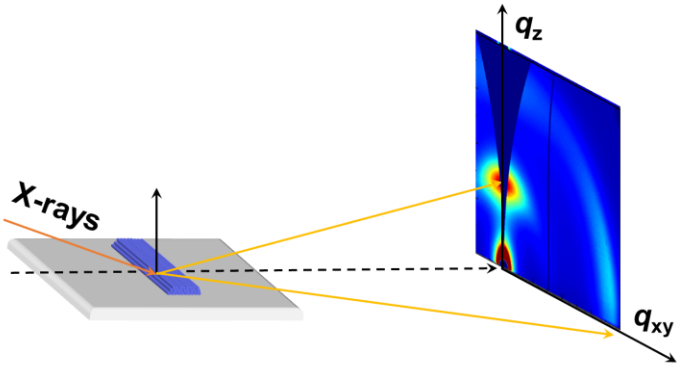

Figure S17 Schematic illustration of GIWAXS measurement setup for BBL fibers. During measurements, fibers were horizontally aligned on the substrate surface, with their longitudinal axes oriented perpendicular to the incident X-ray beam.

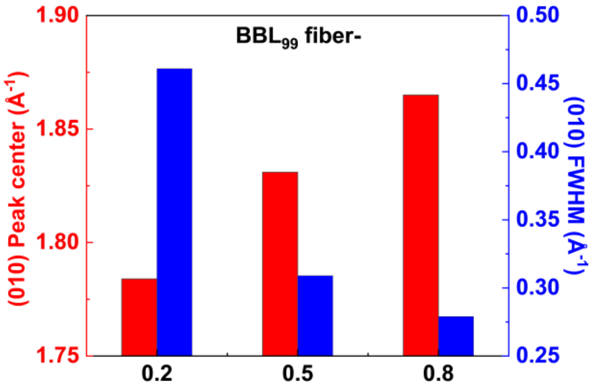

Figure S18 Peak center and FWHM analysis of BBL_{99} fiber.

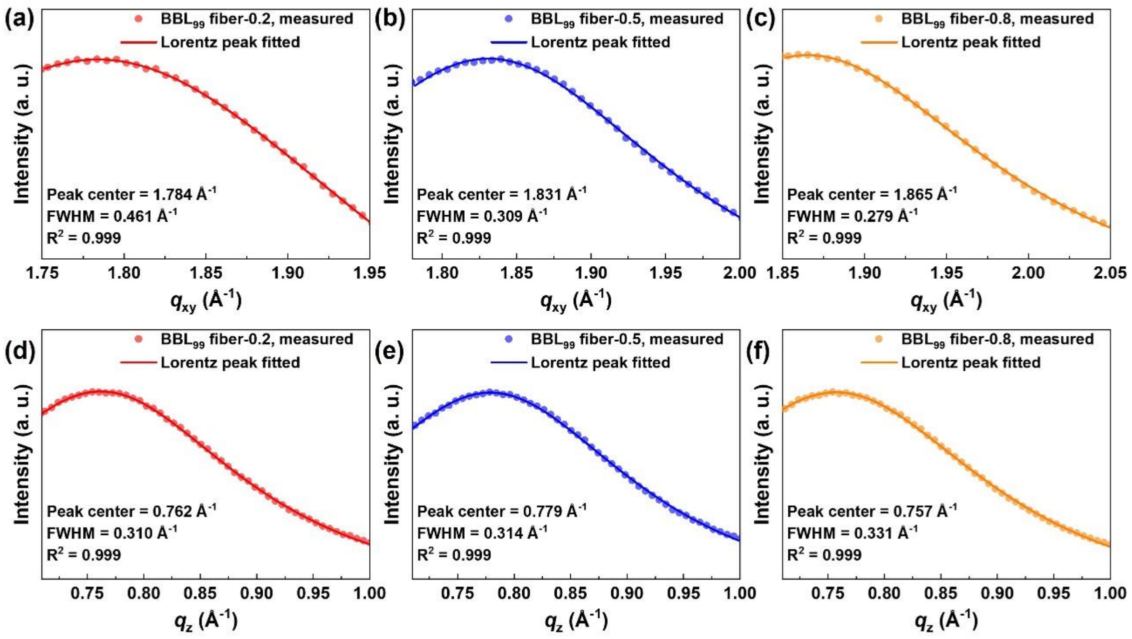

Figure S19 GIWAXS analysis of BBL_{99} fiber.

(a-c) π–π stacking (010) diffraction analysis of BBL_{99} fiber-0.2 (a), BBL_{99} fiber-0.5 (b), and BBL_{99} fiber-0.8 (c). (d-e) Lamellar (100) peak analysis of BBL_{99} fiber-0.2 (d), BBL_{99} fiber-0.5 (e), and BBL_{99} fiber-0.8 (f).

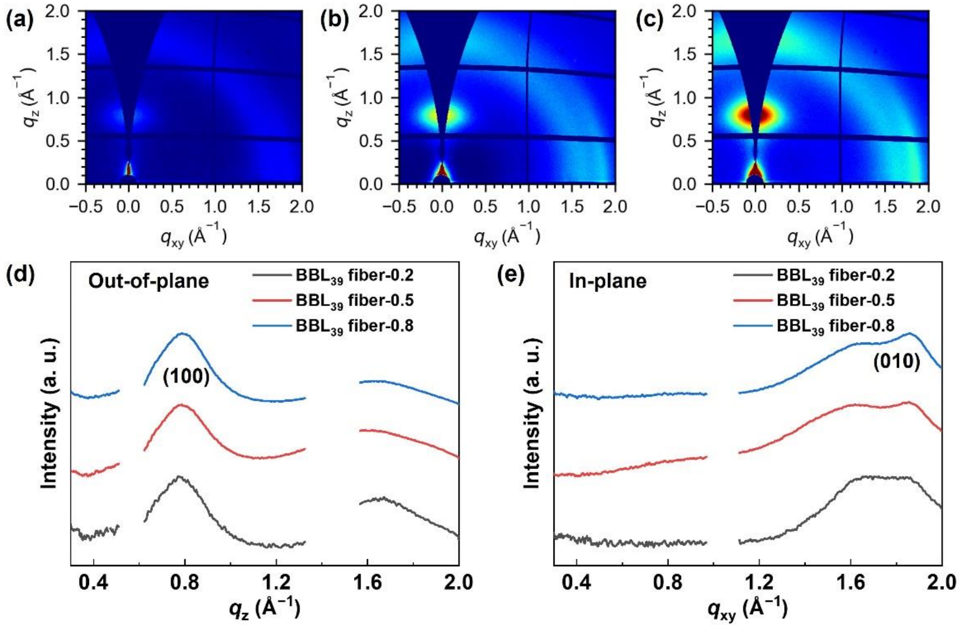

Figure S20 GIWAXS analysis of BBL_{39} fiber.

GIWAXS patterns of BBL_{39} fiber-0.2 (a), BBL_{39} fiber-0.5 (b), and BBL_{39} fiber-0.8 (c). (d,e) In-plane (d) and out-of-plane (e) GIWAXS line cuts of BBL_{39} fiber-0.2, BBL_{39} fiber-0.5, and BBL_{39} fiber-0.8.

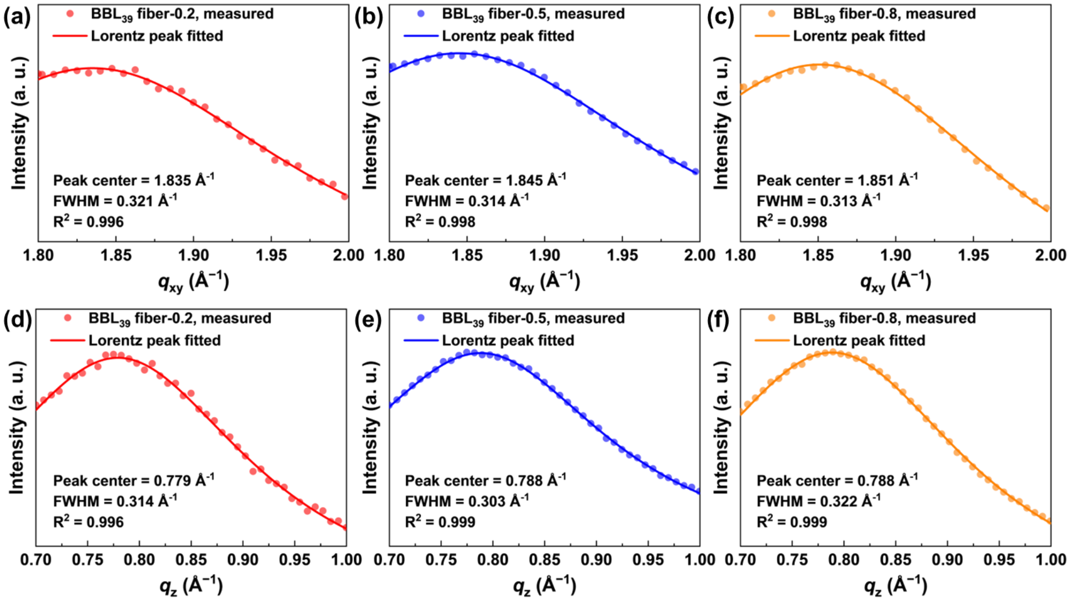

Figure S21 GIWAXS analysis of BBL_{39} fiber.

(a-c) π–π stacking (010) diffraction analysis of BBL_{39} fiber-0.2 (a), BBL_{39} fiber-0.5 (b), and BBL_{39} fiber-0.8 (c). (d-f) Lamellar (100) peak analysis of BBL_{39} fiber-0.2 (d), BBL_{39} fiber-0.5 (e), and BBL_{39} fiber-0.8 (f).

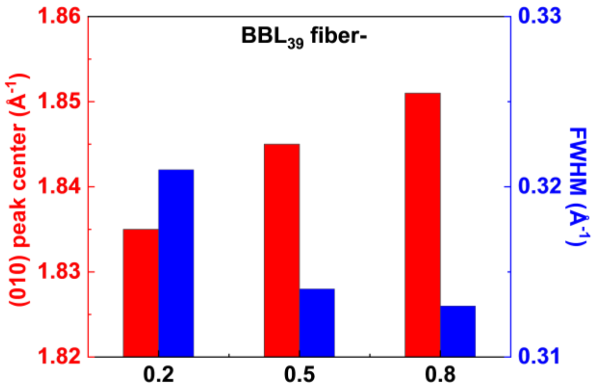

Figure S22 Peak center and FWHM analysis of BBL$_{39}$ fiber.

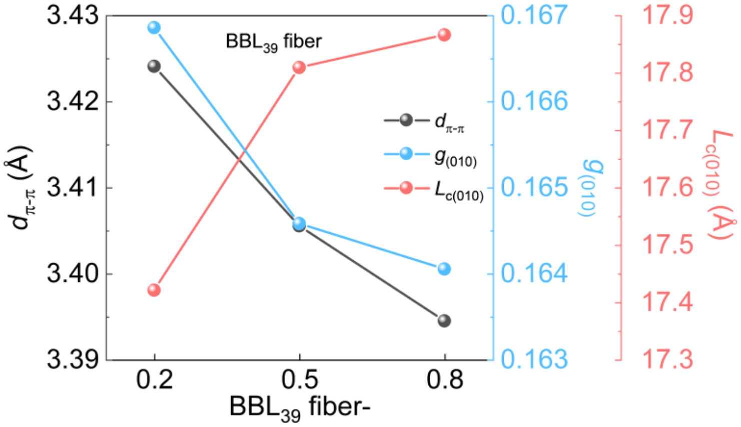

Figure S23 $d_{\pi-\pi}$, $L_{c(010)}$, and $g_{(010)}$ of BBL$_{39}$ fibers.

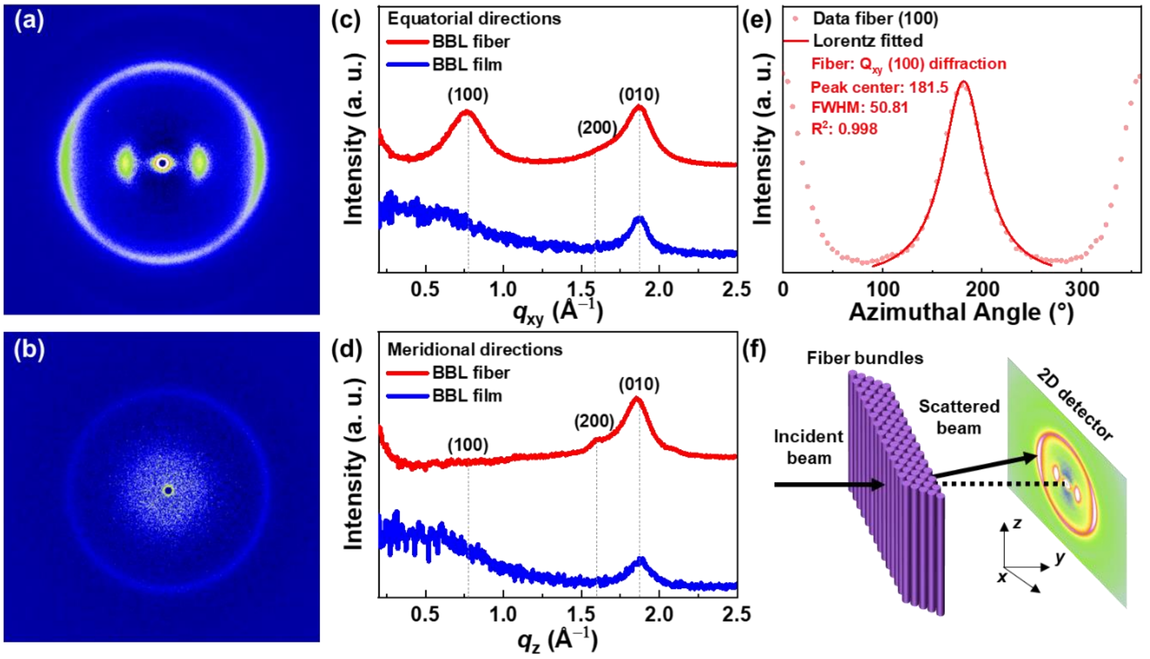

Figure S24 WAXS patterns of BBL99 fiber and film.

(a, b) 2D WAXS patterns of BBL99 fiber (a) and BBL99 film (b). (c, d) Equatorial (c) and meridional (d) directions WAXS line cuts of BBL fiber and film. (e) Azimuthal profiles of (100) reflections were obtained from the WAXS diagram of BBL99 fiber. (f) Schematic diagram of fiber WAXS measurement.

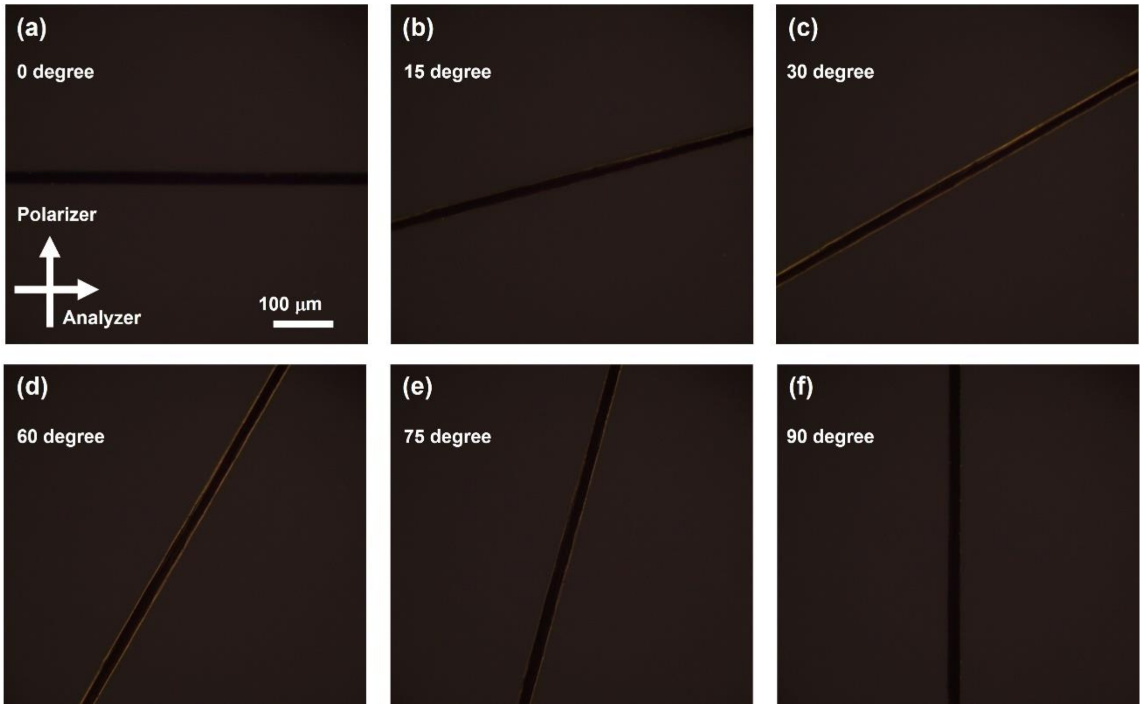

Figure S25 POM images of the BBL_{99} fiber.

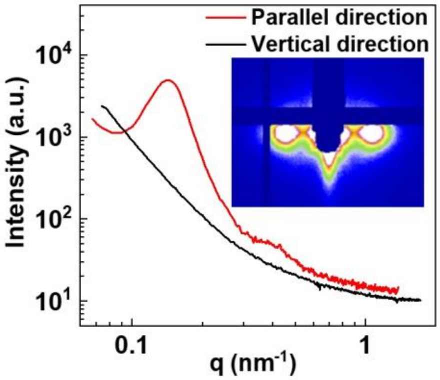

Figure S26 SAXS of BBL_{99} fiber-0.8.

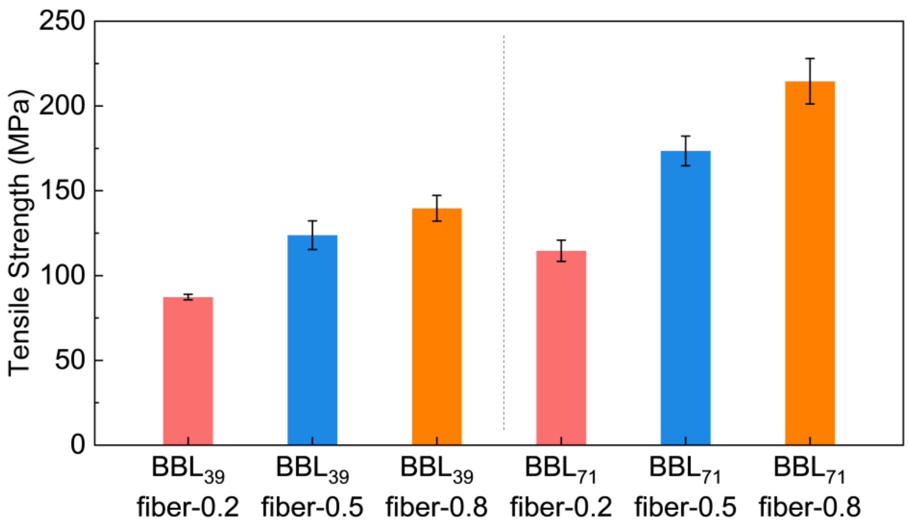

Figure S27 Tensile strength of BBL_{39} fibers and BBL_{71} fibers.

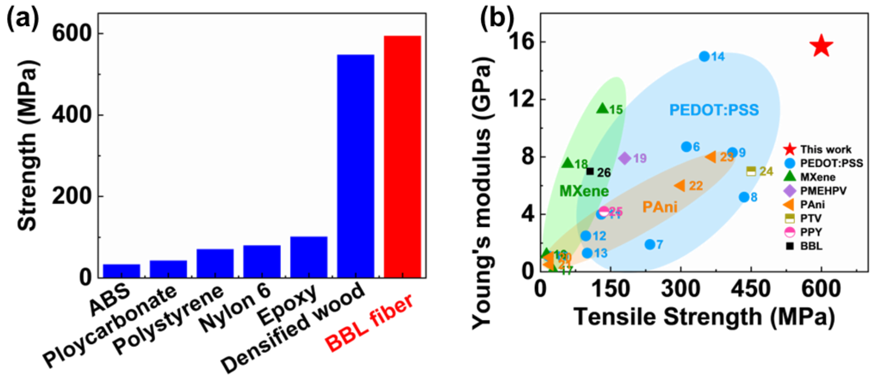

Figure S28 Mechanical properties of semiconductor fibers.

(a) Comparison of the tensile strength of BBL fibers (595.1 MPa) with other broadly applied polymer-based materials and Hu's densified wood. (b) Compares Young's modulus and tensile strength of manufactured BBL fibers with MXene composite fibers, PEDOT:PSS fibers, and other conjugate polymer fibers fabricated in previous studies **[7–27]**. The Ashby plot indicated that wet-spun pure BBL fibers outperformed the other mentioned fibers regarding Young's modulus and tensile strength, implying that the BBL fibers showed superior mechanical properties.

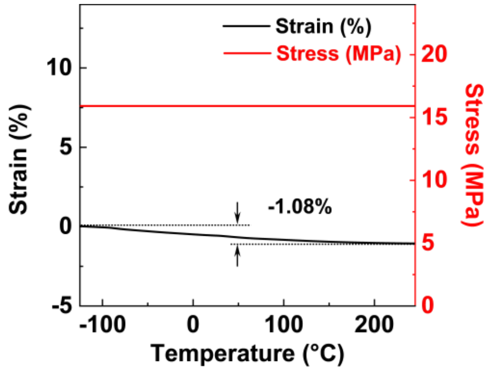

Figure S29 The tensile strain of BBL_{99} fiber-0.8 at a constant applied stress of 16 MPa and variable temperature.

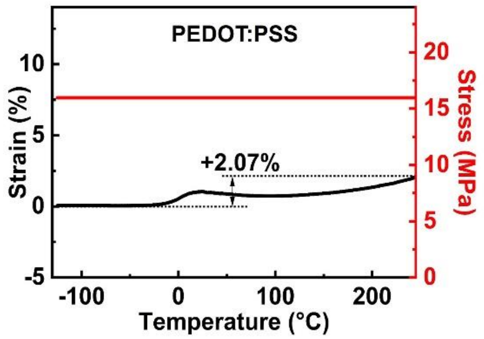

Figure S30 Thermomechanical properties of PEDOT:PSS fiber.

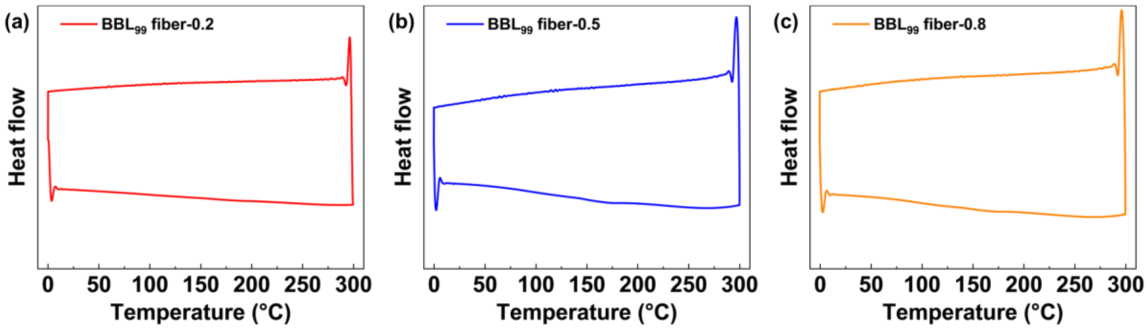

Figure S31 DSC curves of BBL_{99} fibers.

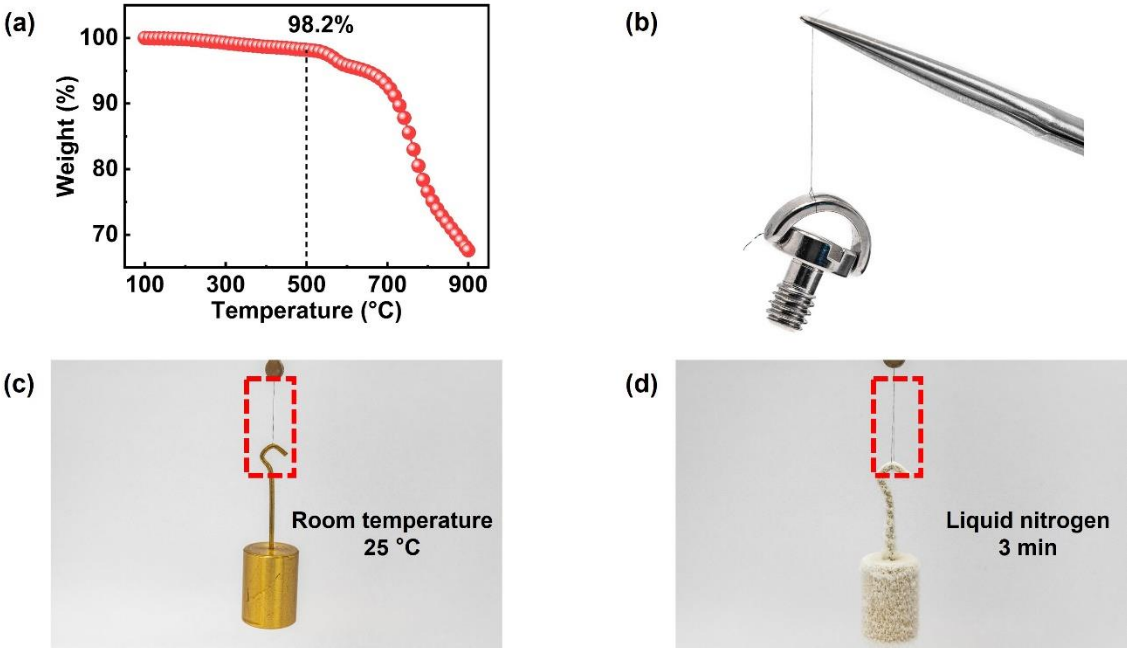

Figure S32 Thermomechanical properties of BBL fibers.

(a) TGA curves of BBL_{99} fibers at 10 K min^{-1} under nitrogen atmosphere. Digital photo of BBL fibers (20 μm) hanging with a bolt (b) and a 10 g weight at room temperature (c) and in liquid nitrogen for 3 minutes (d).

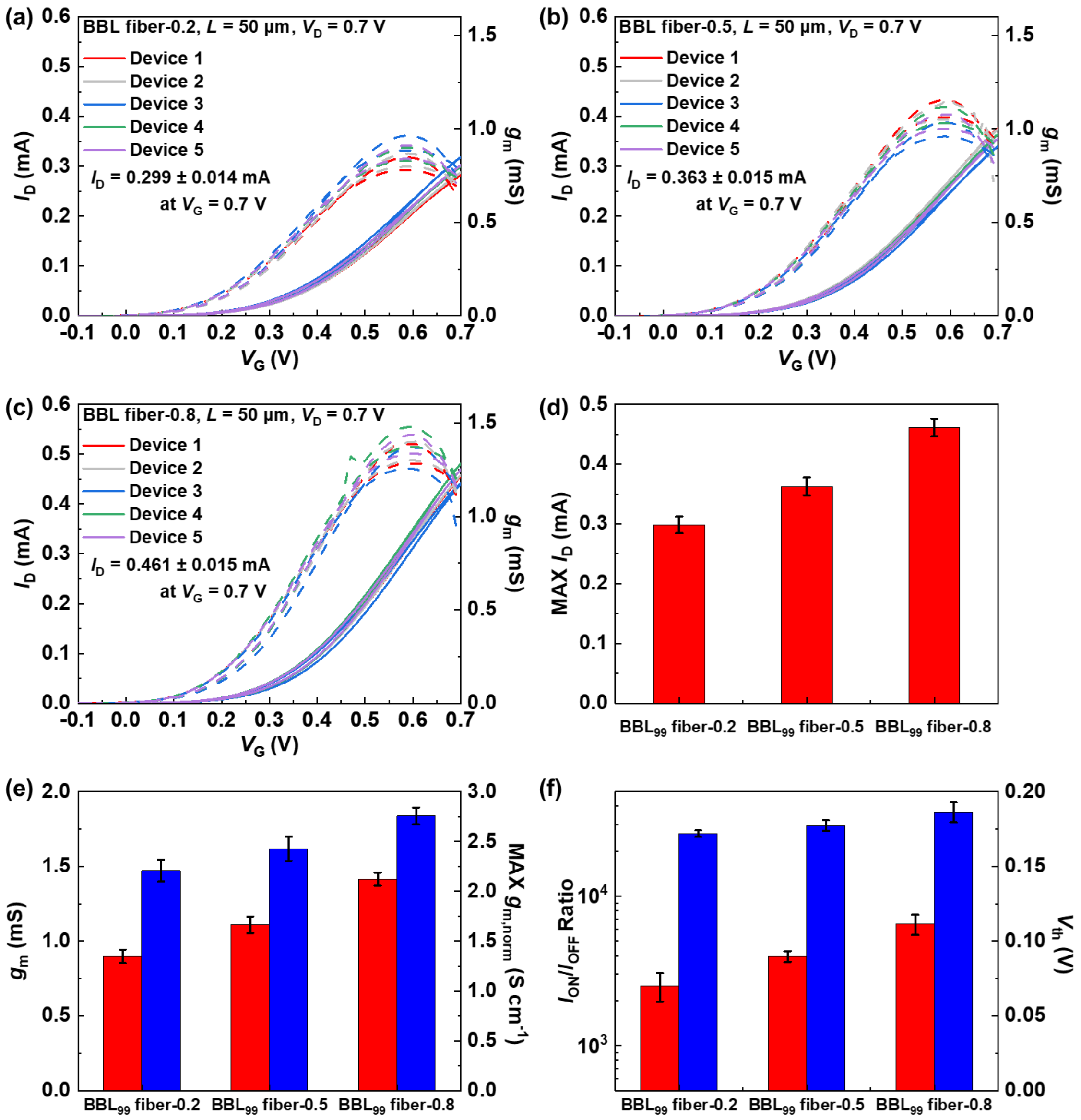

Figure S33 OECT performance of BBL_{99} fiber.

(a-c) Transfer characteristics of five different OECTs based on BBL_{99} fiber-0.2 (a), BBL_{99} fiber-0.5 (b), and BBL_{99} fiber-0.8 (c). (d) All BBL_{99}-based OECTs show reproducibility, with a standard deviation lower than 4.7%. (e) The g_m and MAX $g_{m,norm}$ of BBL_{99} fiber OECTs. (f) The ON/OFF ratio and V_{th} of BBL_{99} fiber OECTs. The error bars indicate standard deviation for five experimental replicates.

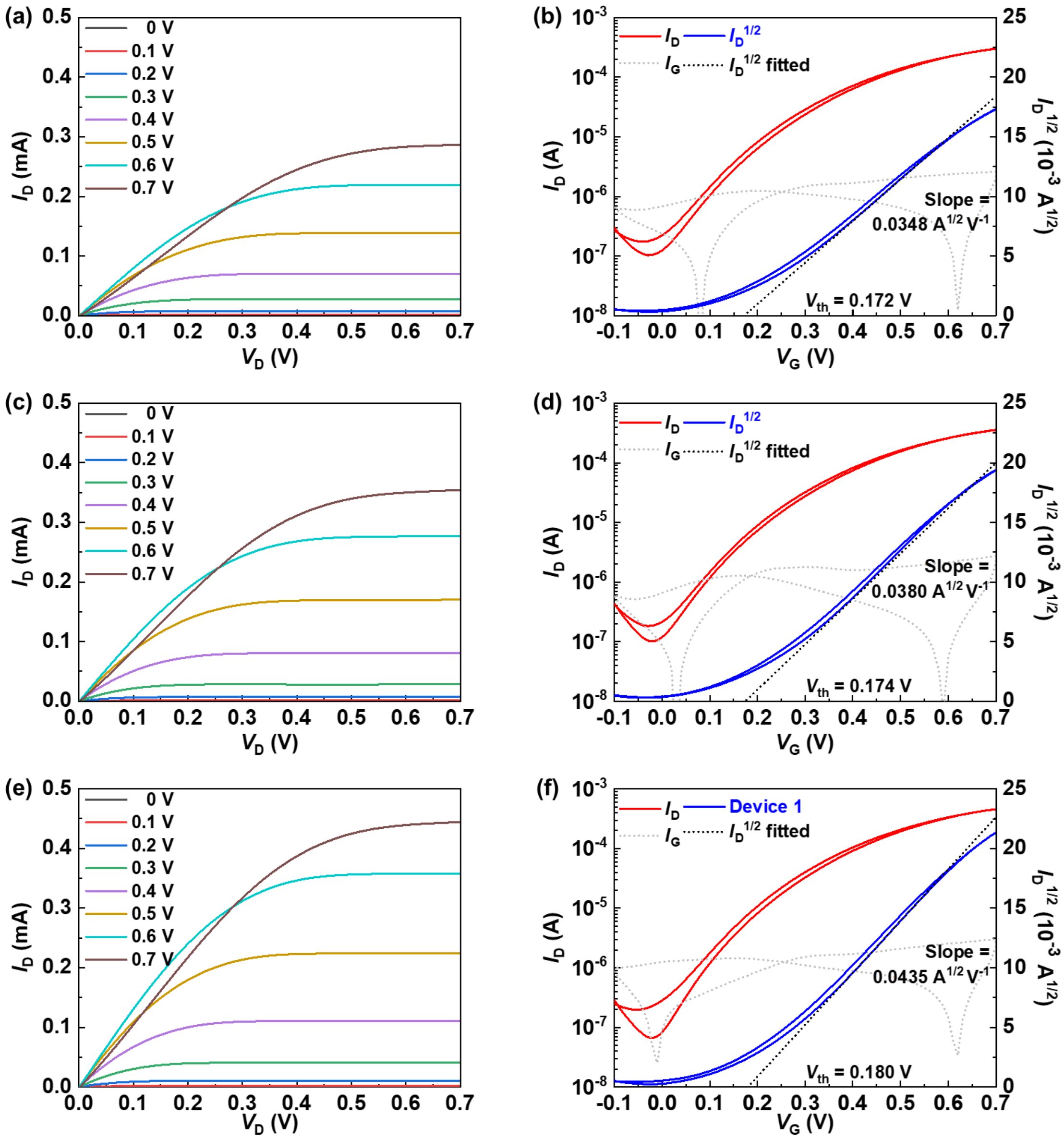

Figure S34 OECT performance of BBL_{99} fiber.

(a-f) Output (a, c, e) and transfer (b, d, f) curves of BBL fiber-0.2 (a, b), BBL fiber-0.5 (c, d), and BBL fiber-0.8 (e, f). Threshold voltages (V_{th}) are also reported. All OECTs have the same channel geometry (L = 50 μm, A was defined by the BBL fiber cross-sectional area).

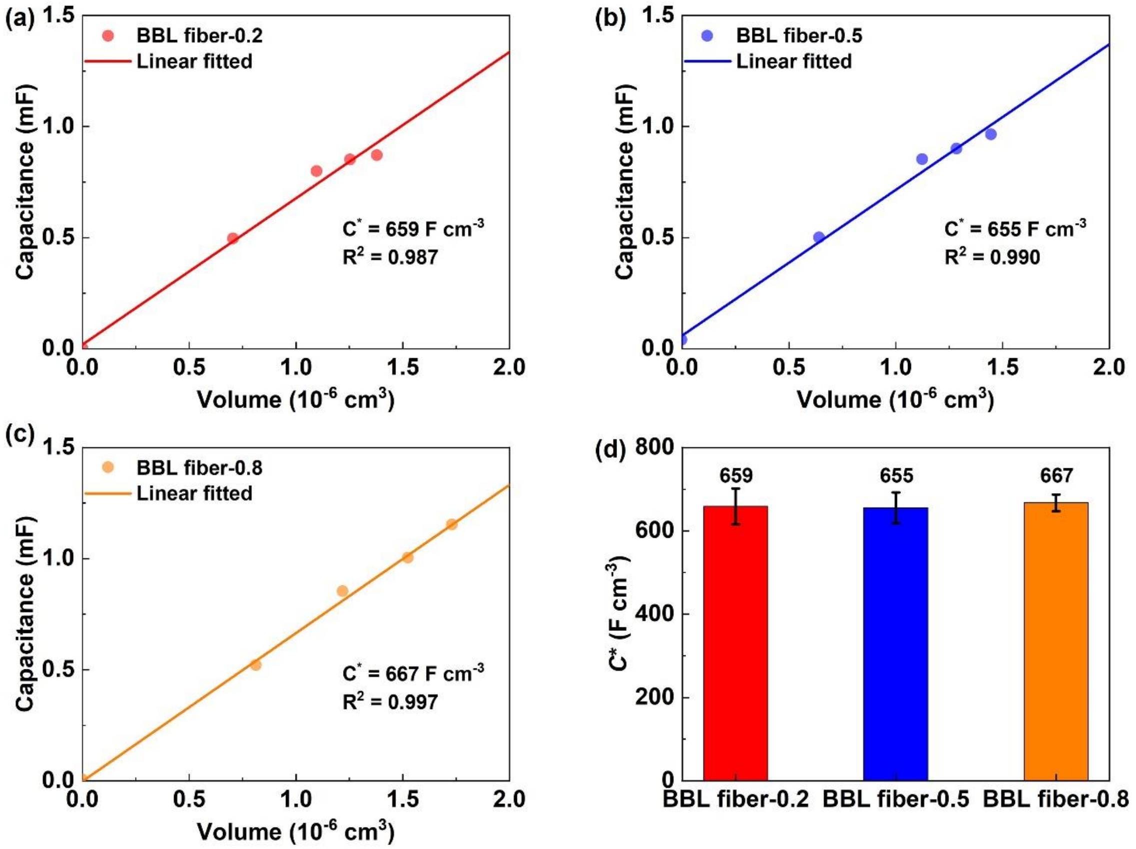

Figure S35 Volumetric capacitance of BBL_{99} fiber.

(a-c) Volume-dependent capacitance of BBL fiber-0.2 (a), BBL fiber-0.5 (b), and BBL fiber-0.8 (c). (d) Summary of volumetric capacitance. The capacitance values were ascertained through analysis of the electrochemical impedance spectroscopy (EIS) spectra, and the volumetric capacitance was determined by employing linear regression on the capacitance data as a function of volume. The error bars indicate standard deviation.

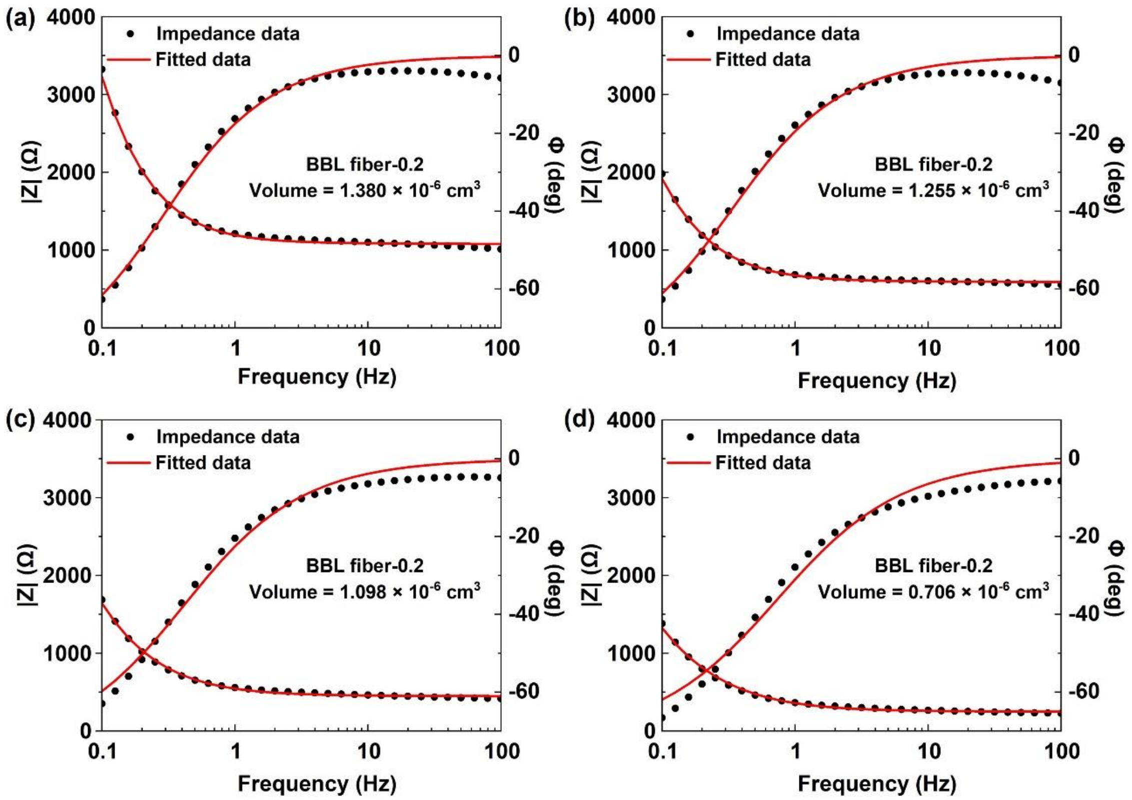

Figure S36 Electrical impedance spectroscopy of BBL_{99} fiber-0.2.

Electrical impedance spectroscopy was carried out on different volumes of BBL_{99} fiber-0.2 placed on a gold electrode as a working electrode. The complex impedance measurements obtained were fitted to the *Rs(Rp||Q)* equivalent circuit.

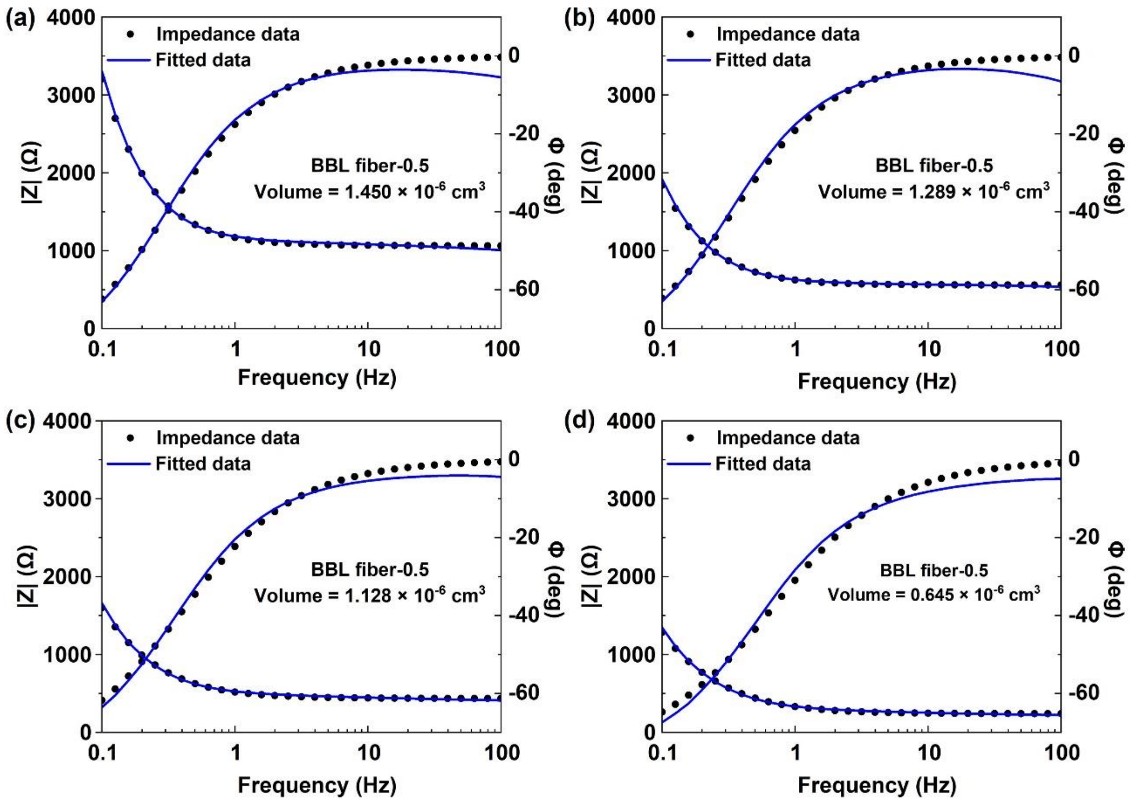

Figure S37 Electrical impedance spectroscopy of BBL_{99} fiber-0.5.

Electrical impedance spectroscopy was carried out on different volumes of BBL_{99} fiber-0.5 placed on a gold electrode as a working electrode. The complex impedance measurements obtained were fitted to the *Rs(Rp||Q)* equivalent circuit.

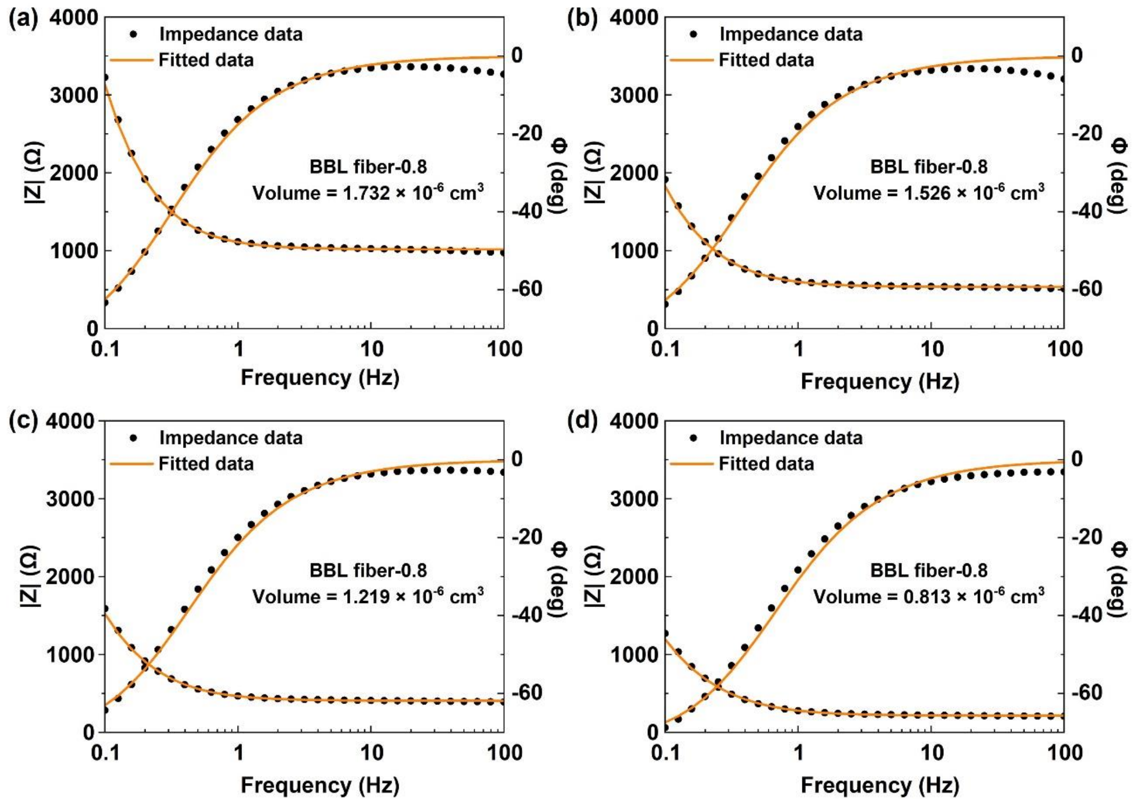

Figure S38 Electrical impedance spectroscopy of BBL_{99} fiber-0.8.

Electrical impedance spectroscopy was carried out on different volumes of BBL_{99} fiber-0.8 placed on a gold electrode as a working electrode. The complex impedance measurements obtained were fitted to the *Rs(Rp||Q)* equivalent circuit.

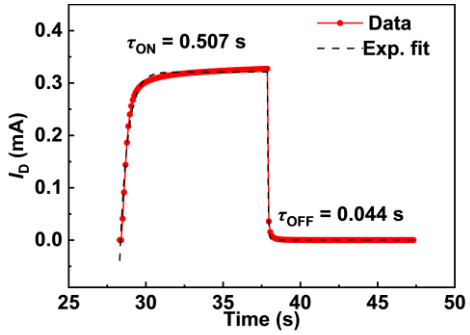

Figure S39 Stability and temporal response of BBL99 fiber OECT.

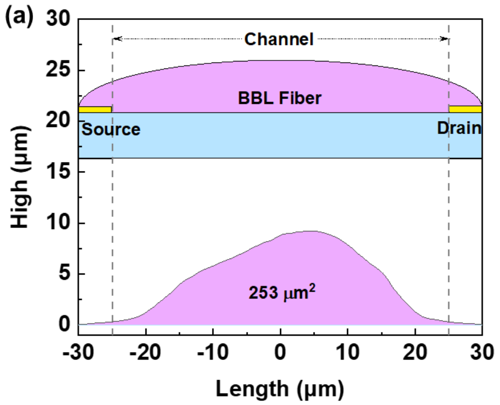

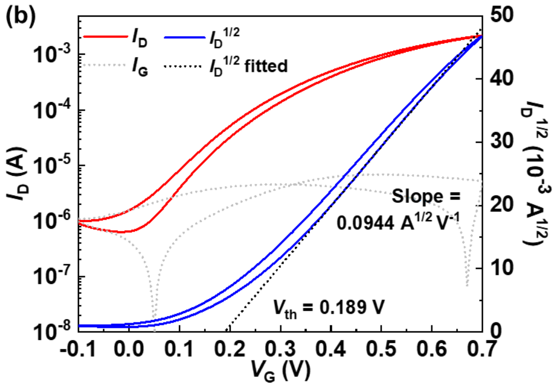

Figure S40 BBL99 fiber radial OECTs.

(a) Cross-sectional area of the BBL fiber-0.8. Top: cross-sectional schematic of the radial OECT. (b) Transfer curves of radial BBL99 fiber-0.8.

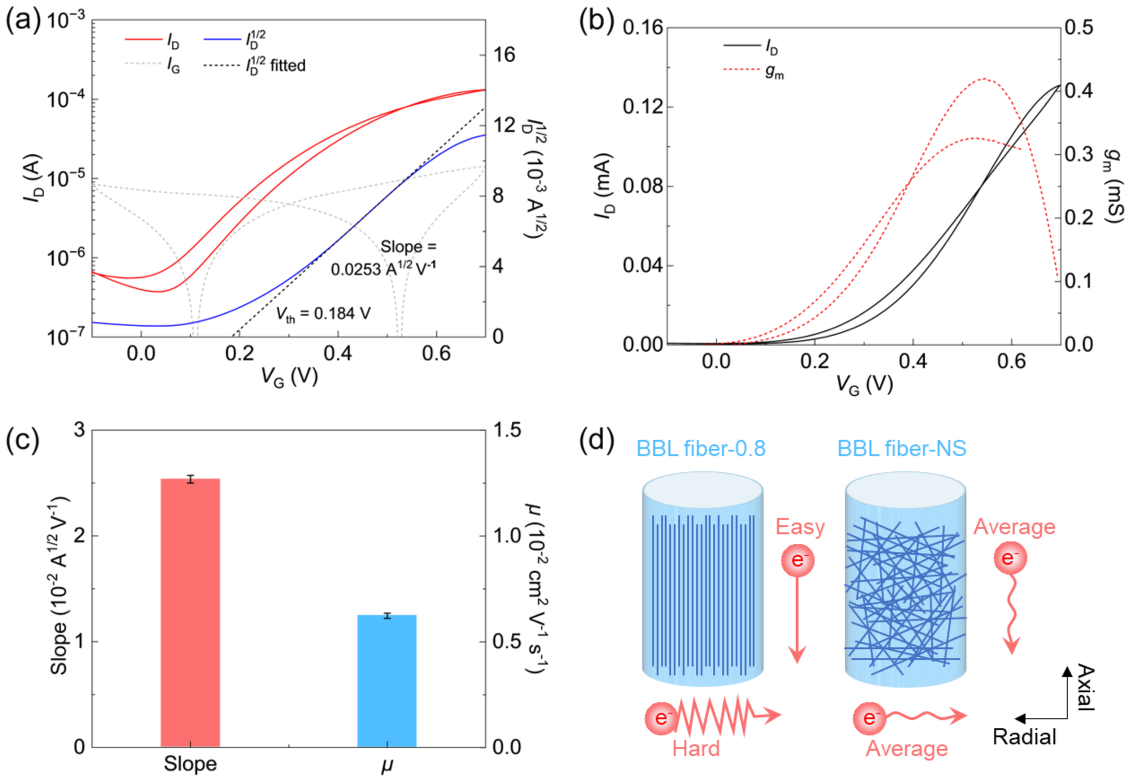

Figure S41 (a) Transfer curves of BBL_{99} fiber-NS OECT. (b) Transfer curve and transconductance curve of BBL_{99} fiber-NS OECT. (c) Carrier mobility of BBL_{99} fiber-NS OECTs. (d) Schematic illustration of charge transport in highly aligned BBL fiber-0.8 (left) and randomly oriented BBL fiber-NS (right). Ordered alignment enhances longitudinal charge transport (easy), while transverse movement is suppressed (hard). In contrast, disordered networks allow moderate transport in all directions (average).

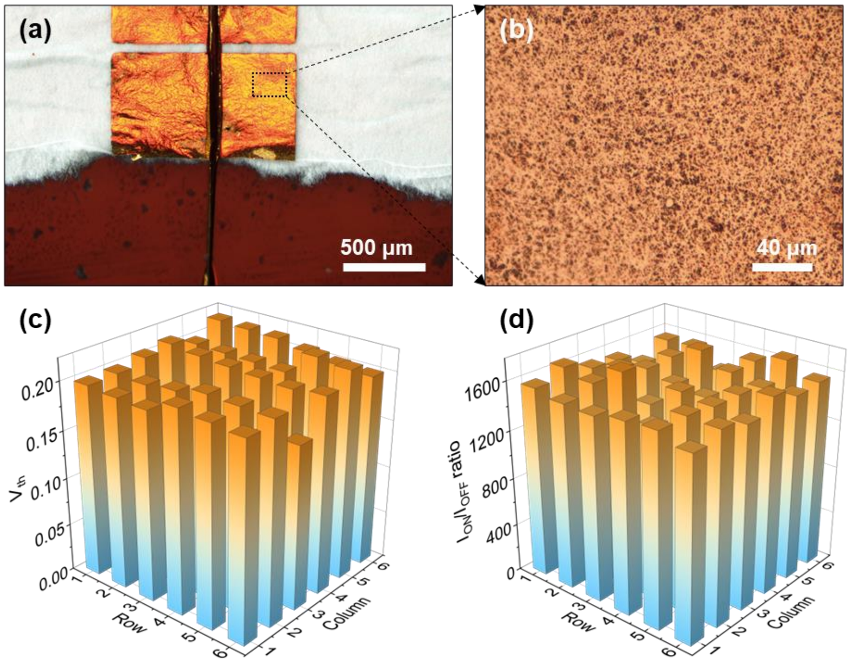

Figure S42 (a) BBL fiber OECT prepared on nanofiber fabric. (b) Fibrous structure of electrode substrate. (c) V_{th} distribution of 6 × 6 BBL fabric OECTs in the array. (d) $I_{\mathrm{ON}}/I_{\mathrm{OFF}}$ ratio distribution of 6 × 6 BBL fabric OECTs in the array.

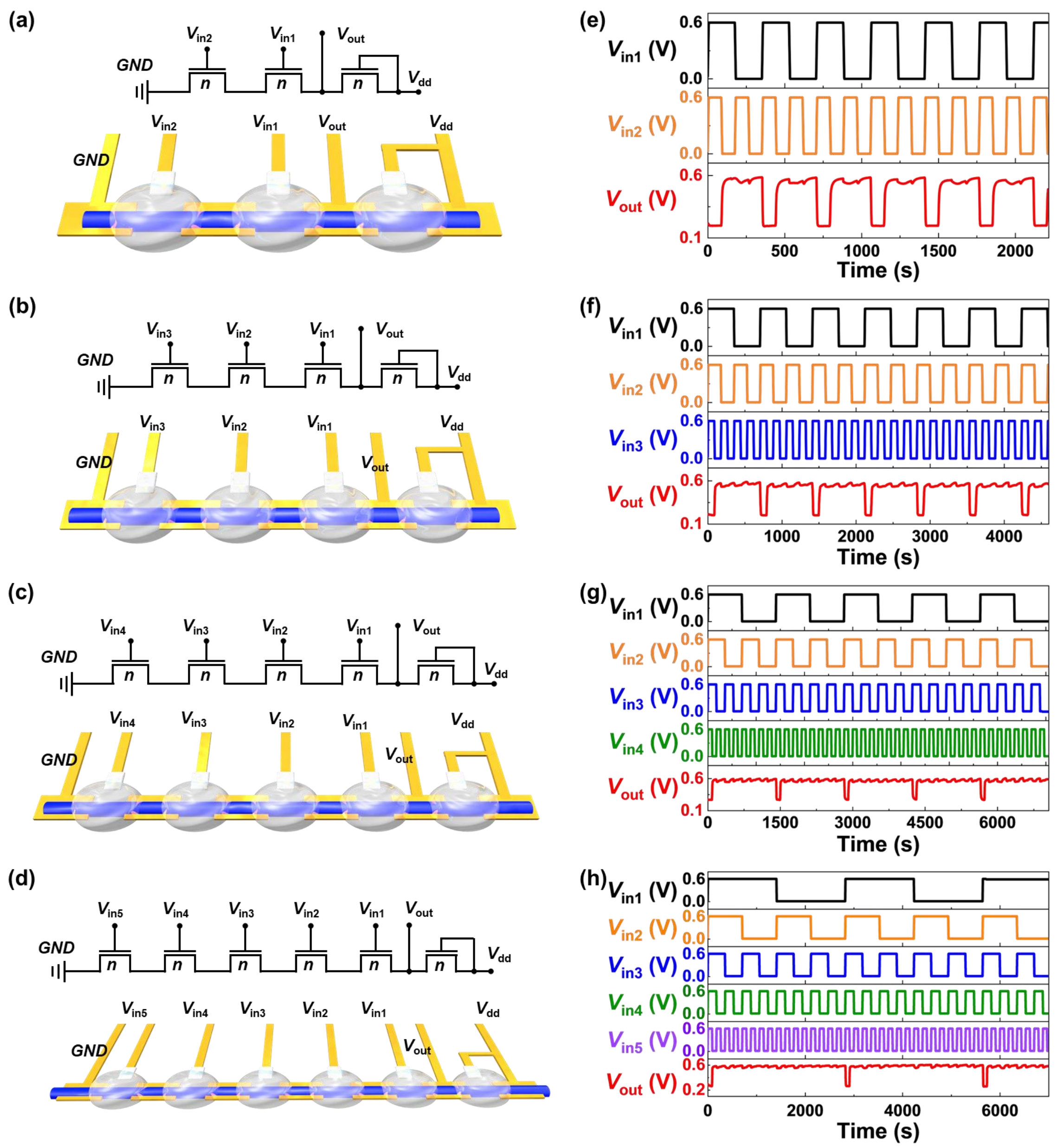

Figure S43 Single semiconductor fiber NAND logic circuits.

A schematic diagram and logic circuit design of the single fiber NAND of 2 (a), 3 (b), 4 (c), and 5 (d) input signals. Output characteristics of the single fiber NAND of 2 (e), 3 (f), 4 (g), and 5 (h) input signals.

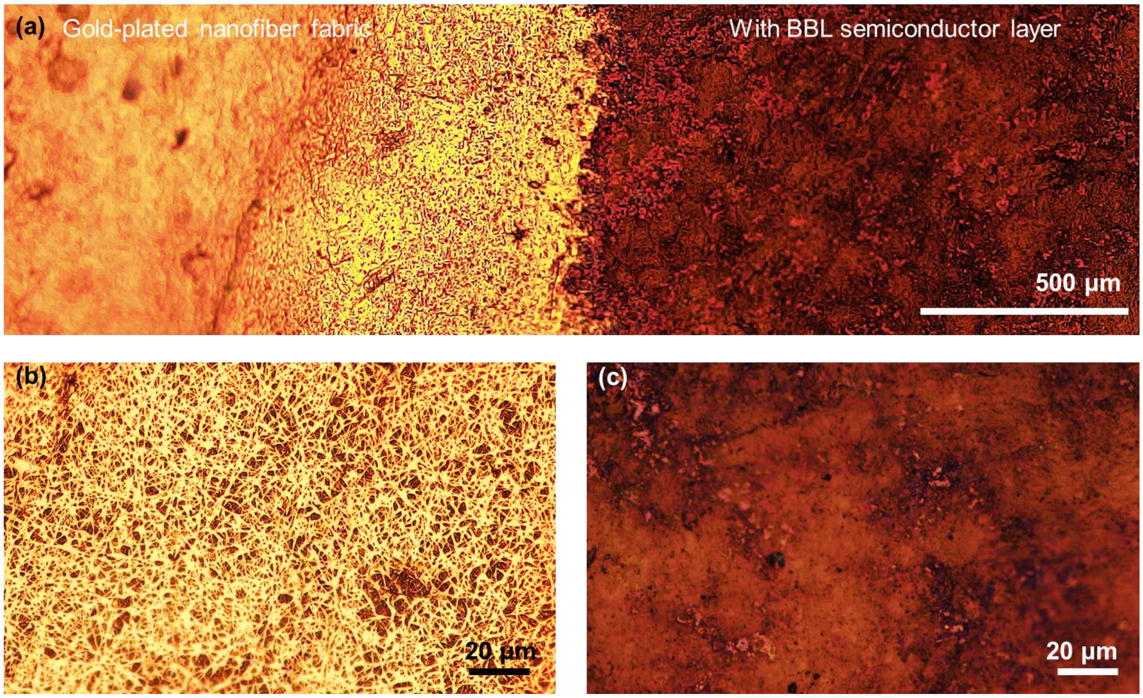

Figure S44 Morphology of fabric-based electrochromic device.

(a) Optical microscopy images of gold-plated nanofiber fabric without (left) and with (right) a composite BBL semiconductor layer. (b) Magnified view of the gold-plated nanofiber fabric, demonstrating the original weave and fiber morphology. (c) Close-up of the gold-plated nanofiber fabric with the BBL composite film, highlighting the changes in surface texture and color due to the presence of the semiconductor.

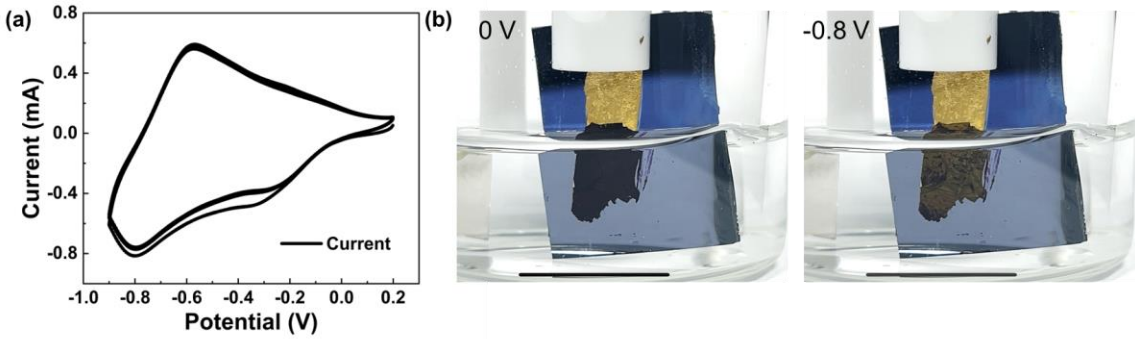

Figure S45 Performance of fabric-based electrochromic device.

(a) Cyclic voltammetry (CV) tests of fabric-based electrochromic devices. (b) Optical images of fabric-based electrochromic devices captured at different color states.

Table S1 Dynamic viscosity and shear stress of BBL_{99}-MSA solutions.

Concentration (mg mL^{-1})	Flow velocity (mL min^{-1})	Density (kg m^{-3})	Dynamic viscosity ($\times 10^{-2}$ Pa s)	Maximum shear stress (Pa)
1	0.1	1481	1.73	36.81
3		1483	3.23	69.25
5		1485	4.74	102.52
7		1487	6.25	136.48
10		1490	8.52	187.88
1	0.2	1481	1.73	73.61
3		1483	3.23	137.66
5		1485	4.74	202.26
7		1487	6.25	267.58
10		1490	8.52	367.53
1	0.3	1481	1.73	110.58
3		1483	3.23	206.45
5		1485	4.74	302.82
7		1487	6.25	399.67
10		1490	8.52	546.72
1	0.4	1481	1.73	147.70
3		1483	3.23	275.43
5		1485	4.74	403.67
7		1487	6.25	532.33
10		1490	8.52	727.03
1	0.5	1481	1.73	184.99
3		1483	3.23	344.54
5		1485	4.74	504.74
7		1487	6.25	665.24
10		1490	8.52	907.86
1	0.6	1481	1.73	222.43
3		1483	3.23	413.82
5		1485	4.74	605.96
7		1487	6.25	798.43
10		1490	8.52	1089.09
1	0.7	1481	1.73	260.02
3		1483	3.23	483.27
5		1485	4.74	707.31
7		1487	6.25	931.73
10		1490	8.52	1270.55
1	0.8	1481	1.73	297.83
3		1483	3.23	552.84
5		1485	4.74	808.81
7		1487	6.25	1065.22
10		1490	8.52	1452.21

Table S2 Average velocities and Reynolds number of BBL_{99}-MSA solutions.

Concentration ($mg\ mL^{-1}$)	Flow velocity ($mL\ min^{-1}$)	Density ($kg\ m^{-3}$)	Average velocity ($\times10^{-1}\ m\ s^{-1}$)	Reynolds number (a.u.)
1	0.1	1481	0.064	1.090
3		1483	0.064	0.587
5		1485	0.064	0.400
7		1487	0.064	0.303
10		1490	0.064	0.222
1	0.2	1481	0.128	2.192
3		1483	0.128	1.175
5		1485	0.128	0.803
7		1487	0.128	0.609
10		1490	0.128	0.447
1	0.3	1481	0.192	3.294
3		1483	0.192	1.763
5		1485	0.192	1.205
7		1487	0.192	0.915
10		1490	0.192	0.672
1	0.4	1481	0.256	4.395
3		1483	0.256	2.351
5		1485	0.256	1.608
7		1487	0.256	1.221
10		1490	0.256	0.896
1	0.5	1481	0.320	5.496
3		1483	0.320	2.938
5		1485	0.320	2.010
7		1487	0.320	1.526
10		1490	0.320	1.121
1	0.6	1481	0.385	6.596
3		1483	0.384	3.526
5		1485	0.385	2.411
7		1487	0.385	1.831
10		1490	0.385	1.345
1	0.7	1481	0.449	7.696
3		1483	0.448	4.113
5		1485	0.449	2.813
7		1487	0.449	2.137
10		1490	0.449	1.569
1	0.8	1481	0.513	8.796
3		1483	0.512	4.700
5		1485	0.513	3.215
7		1487	0.513	2.442
10		1490	0.513	1.794

Table S3 Concentration of the polymer and dynamic viscosity of the solution.

Solute	Solvent	Concentration (mg mL^{-1})	Density of the solution (kg m^{-3})	Kinematic viscosity (mm^2 s^{-1})	Dynamic viscosity ($\times 10^{-2}$ Pa s)
BBL_{99}	MSA	5.0	1485.0	31.9	4.74
BBL_{39}	MSA	10.1	1490.0	31.5	4.70
BBB	MSA	24.1	1504.1	31.4	4.73
PBFDO	DMSO	7.6	1106.9	42.8	4.73
PEDOT:PSS	H_2O	6.8	1006.8	47.5	4.78
p(g2T-T)	MSA	24.8	1504.8	11.4	1.71

Table S4 Summary of BBL_{99} fiber OECT.

Material	L (μm)	Fiber diameter (μm)	A (μm^2)	$g_{m,norm}$ (S cm^{-1})	V_{th} (V)	I_{ON}/I_{OFF} ($\times 10^3$)
BBL_{99} fiber-0.2	50	16.10 ± 0.09	203.48	2.21 ± 0.11	0.17 ± 0.01	2.52 ± 0.54
BBL_{99} fiber-0.5	50	17.06 ± 0.13	228.48	2.43 ± 0.12	0.177 ± 0.01	3.96 ± 0.33
BBL_{99} fiber-0.8	50	18.08 ± 0.11	256.85	2.76 ± 0.09	0.186 ± 0.01	6.51 ± 0.10

Table S5 Summary of BBL_{99} fiber OECT performance

Material	L (μm)	Fiber diameter (μm)	μC^* (F cm^{-1} V^{-1} s^{-1})	C^* (F cm^{-3})	μ (cm^2 V^{-1} s^{-1})
BBL_{99} fiber-0.2	50	16.10 ± 0.09	5.91 ± 0.179	659 ± 43	$(8.98 \pm 0.65) \times 10^{-3}$
BBL_{99} fiber-0.5	50	17.06 ± 0.13	6.59 ± 0.32	655 ± 27	$(1.01 \pm 0.08) \times 10^{-2}$
BBL_{99} fiber-0.8	50	18.08 ± 0.11	7.66 ± 0.48	667 ± 20	$(1.15 \pm 0.08) \times 10^{-2}$

Table S6 The electrical properties in fiber-based OECTs

Ref *No.*	Channel	On/Off ratio	Drive (V)	g_m (mS)	Type
Ref. S[7]	PEDOT:PSS	10^3	1	1	Depletion
Ref. S[28]	PEDOT:PSS	10^2	1	~0.74	Depletion
Ref. S[29]	PPy/PVA/PE	~10^2	3	-	Depletion
Ref. S[30]	PPy	10^4	2	-	Depletion
Ref. S[31]	CNT	10^2	1	1.35	Depletion
Ref. S[32]	PPy/Graphene	10^2	2	-	Depletion
Ref. S[33]	PAni	10^3	0.6	0.06	Depletion
This work	BBL	10^3	0.6	1.29	Enhancement

Supplementary Movies

Movie S1 (separate file). Continuous liquid crystal spinning of BBL semiconducting fibers.

Movie S2 (separate file). Thermo-mechanical properties of BBL semiconductor fibers.

SI References

1. Van Deusen RL. Benzimidazo-benzophenanthroline polymers. *J. Polym. Sci. [B]* 1966; **4**: 211–14.
2. Van Deusen RL, Goins OK, Sicree AJ. Thermally stable polymers from 1,4,5,8-naphthalenetetracarboxylic acid and aromatic tetraamines. *J. Polym. Sci. [A1]* 1968; **6**: 1777–93.
3. Lin D, Li T, Li R, et al. Structures and properties of polyimide fibers prepared via gel spinning induced by chemical imidization. *Polymer* 2022; **238**: 124377.
4. Flory PJ. Molecular theory of liquid crystals. *Liq. Cryst. Polym. I* 1984; 1–36.
5. Roberts MF, Jenekhe SA. Lewis acid coordination complexes of polymers. 1. Boron chloride, aluminum chloride and gallium chloride complexes of poly(p-phenylenebenzobisthiazole). *Chem. Mater.* 1993; **5**: 1744–54.
6. Roberts MF, Jenekhe SA. Lewis acid coordination complexes of polymers: 3. Poly(benzobisimidazobenzophenanthroline) ladder and semiladder polymers. *Polymer* 1994; **35**: 4313–25.
7. Kim Y, Noh H, Paulsen BD, et al. Strain-engineering induced anisotropic crystallite orientation and maximized carrier mobility for high-performance microfiber-based organic bioelectronic devices. *Adv. Mater.* 2021; **33**: 2007550.
8. Sarabia-Riquelme R, Andrews R, Anthony JE, et al. Highly conductive wet-spun PEDOT:PSS fibers for applications in electronic textiles. *J. Mater. Chem. C* 2020; **8**: 11618–30.
9. Kim Y, Lund A, Noh H, et al. Robust PEDOT:PSS Wet-Spun Fibers for Thermoelectric Textiles. *Macromol. Mater. Eng.* 2020; **305**: 1900749.
10. Zhang J, Seyedin S, Qin S, et al. Highly Conductive Ti3C2Tx MXene Hybrid Fibers for Flexible and Elastic Fiber-Shaped Supercapacitors. *Small* 2019; **15**: 1804732.
11. Zhang J, Seyedin S, Qin S, et al. Fast and scalable wet-spinning of highly conductive PEDOT:PSS fibers enables versatile applications. *J. Mater. Chem. A* 2019; **7**: 6401–10.
12. Wang Z, Qin S, Seyedin S, et al. High-Performance Biscrolled MXene/Carbon Nanotube Yarn Supercapacitors. *Small* 2018; **14**: 1802225.
13. Kim Y, Lim T, Kim C-H, et al. Organic electrochemical transistor-based channel dimension-independent single-strand wearable sweat sensors. *NPG Asia Mater.* 2018; **10**: 1086–95.
14. Yang Q, Xu Z, Fang B, et al. MXene/graphene hybrid fibers for high performance flexible supercapacitors. *J. Mater. Chem. A* 2017; **5**: 22113–19.
15. Seyedin S, Yanza ERS, Razal JM. Knittable energy storing fiber with high volumetric performance made from predominantly MXene nanosheets. *J. Mater. Chem. A* 2017; **5**: 24076–82.
16. Zhou J, Li EQ, Li R, et al. Semi-metallic, strong and stretchable wet-spun conjugated polymer microfibers. *J. Mater. Chem. C* 2015; **3**: 2528–38.

17. Jalili R, Razal JM, Innis PC, et al. One-Step Wet-Spinning Process of Poly(3,4-ethylenedioxythiophene):Poly(styrenesulfonate) Fibers and the Origin of Higher Electrical Conductivity. *Adv. Funct. Mater.* 2011; **21**: 3363–70.
18. Okuzaki H, Harashina Y, Yan H. Highly conductive PEDOT/PSS microfibers fabricated by wet-spinning and dip-treatment in ethylene glycol. *Eur. Polym. J.* 2009; **45**: 256–61.
19. Foroughi J, Spinks GM, Wallace GG, et al. Production of polypyrrole fibres by wet spinning. *Synth. Met.* 2008; **158**: 104–7.
20. Bowman D, Mattes BR. Conductive Fibre Prepared From Ultra-High Molecular Weight Polyaniline for Smart Fabric and Interactive Textile Applications. *Synth. Met.* 2005; **154**: 29–32.
21. Okuzaki H, Ishihara M. Spinning and Characterization of Conducting Microfibers. *Macromol. Rapid Commun.* 2003; **24**: 261–64.
22. Andreatta A, Smith P. Processing of conductive polyaniline-UHMW polyethylene blends from solutions in non-polar solvents. *Synth. Met.* 1993; **55**: 1017–22.
23. Motamedi F, Ihn KJ, Ni Z, et al. Fibres of poly (methoxy-2-ethyl-hexyloxy) phenylenevinylene prepared from the soluble, fully conjugated polymer. *Polymer* 1992; **33**: 1102–4.
24. Scherr EM, MacDiarmid AG, Manohar SK, et al. Polyaniline: Oriented films and fibers. *Synth. Met.* 1991; **41**: 735–38.
25. Tokito S, Smith P, Heeger AJ. Mechanical and electrical properties of poly-(2,5-thienylene vinylene) fibers. *Synth. Met.* 1990; **36**: 183–94.
26. Andreatta A, Cao Y, Chiang JC, et al. Electrically-conductive fibers of polyaniline spun from solutions in concentrated sulfuric acid. *Synth. Met.* 1988; **26**: 383–89.
27. Wang X, Zhang Z, Li P, et al. Ultrastable N-Type Semiconducting Fiber Organic Electrochemical Transistors for Highly Sensitive Biosensors. *Adv. Mater.* 2024; **36**: 2400287.
28. Alarcon-Espejo P, Sarabia-Riquelme R, Matrone GM, et al. High-Hole-Mobility Fiber Organic Electrochemical Transistors for Next-Generation Adaptive Neuromorphic Bio-Hybrid Technologies. *Adv. Mater.* 2024; **36**: 2305371.
29. Qing X, Wang Y, Zhang Y, et al. Wearable fiber-based organic electrochemical transistors as a platform for highly sensitive dopamine monitoring. *ACS Appl. Mater. Interfaces* 2019; **11**: 13105–13.
30. Wang Y, Zhou Z, Qing X, et al. Ion sensors based on novel fiber organic electrochemical transistors for lead ion detection. *Anal. Bioanal. Chem.* 2016; **408**: 5779–87.
31. Wu X, Feng J, Deng J, et al. Fiber-shaped organic electrochemical transistors for biochemical detections with high sensitivity and stability. *Sci. China Chem.* 2020; **63**: 1281–88.
32. Wang Y, Qing X, Zhou Q, et al. The woven fiber organic electrochemical transistors based on polypyrrole nanowires/reduced graphene oxide composites for glucose sensing. *Biosens. Bioelectron.* 2017; **95**: 138–45.
33. Fang B, Yan J, Chang D, et al. Scalable production of ultrafine polyaniline fibres for tactile organic electrochemical transistors. *Nat. Commun.* 2022; **13**: 2101.
